# Ca^2+^/Cation Antiporters (CaCA): Identification, Characterization and Expression Profiling in Bread Wheat (*Triticum aestivum* L.)

**DOI:** 10.3389/fpls.2016.01775

**Published:** 2016-11-28

**Authors:** Mehak Taneja, Shivi Tyagi, Shailesh Sharma, Santosh Kumar Upadhyay

**Affiliations:** ^1^Department of Botany, Panjab UniversityChandigarh, India; ^2^National Agri-Food Biotechnology InstituteMohali, India

**Keywords:** abiotic, biotic, Ca^2+^/cation antiporters, TaCAX, TaCCX, TaNCL, TaMHX, *Triticum aestivum*

## Abstract

The Ca^2+^/cation antiporters (CaCA) superfamily proteins play vital function in Ca^2+^ ion homeostasis, which is an important event during development and defense response. Molecular characterization of these proteins has been performed in certain plants, but they are still not characterized in *Triticum aestivum* (bread wheat). Herein, we identified 34 TaCaCA superfamily proteins, which were classified into TaCAX, TaCCX, TaNCL, and TaMHX protein families based on their structural organization and evolutionary relation with earlier reported proteins. Since the *T. aestivum* comprises an allohexaploid genome, *TaCaCA* genes were derived from each A, B, and D subgenome and homeologous chromosome (HC), except chromosome-group 1. Majority of genes were derived from more than one HCs in each family that were considered as homeologous genes (HGs) due to their high similarity with each other. These HGs showed comparable gene and protein structures in terms of exon/intron organization and domain architecture. Majority of TaCaCA proteins comprised two Na_Ca_ex domains. However, TaNCLs consisted of an additional EF-hand domain with calcium binding motifs. Each TaCaCA protein family consisted of about 10 transmembrane and two α-repeat regions with specifically conserved signature motifs except TaNCL, which had single α-repeat. Variable expression of most of the *TaCaCA* genes during various developmental stages suggested their specified role in development. However, constitutively high expression of a few genes like *TaCAX1-A* and *TaNCL1-B* indicated their role throughout the plant growth and development. The modulated expression of certain genes during biotic (fungal infections) and abiotic stresses (heat, drought, salt) suggested their role in stress response. Majority of *TaCCX* and *TaNCL* family genes were found highly affected during various abiotic stresses. However, the role of individual gene needs to be established. The present study unfolded the opportunity for detail functional characterization of TaCaCA proteins and their utilization in future crop improvement programs.

## Introduction

Calcium ion (Ca^2+^) is a vital element in plants due to its roles as an essential nutrient and secondary messenger (Hirschi, 2004; Case et al., 2007). It is involved in signal transduction in response to various internal and/or external stimuli (Poovaiah and Reddy, 1993; Dodd et al., 2010; Spalding and Harper, 2011). The concentration of Ca^2+^ increases during perturbation of stimuli, which get recognized by calcium binding proteins or sensor proteins. These proteins further transfer the signal downstream to start phosphorylation cascade that ultimately lead to the regulation of gene expression (Tuteja and Mahajan, 2007). The elevation in Ca^2+^ concentration inside the cell is also reported in response to various growth regulators, plant nutrients, pathogens and abiotic stresses, which established the role of calcium signaling during development and stress responses in plants (Tuteja and Sopory, 2008; Kader and Lindberg, 2010; Zhang et al., 2014a).

The modulation in Ca^2+^ concentration across the cell membrane is basically mediated by three classes of transporters- Ca^2+^-ATPases (PMCAs), Ca^2+^ permeable channels, and Ca^2+^/cation antiporters (CaCAs), which function in combination of each other (Haug-Collet et al., 1999; Sanders et al., 1999; Axelsen and Palmgren, 2001). The CaCA superfamily proteins are reported in diverse group of organisms from bacteria to higher plants and animals as well. These proteins usually facilitate the efflux of Ca^2+^ against concentration gradient across the membrane, and influx of monovalent cations like H^+^, Na^+^, or K^+^ in exchange (Saier et al., 1999; Cai and Lytton, 2004a; Emery et al., 2012; Pittman and Hirschi, 2016a). CaCA proteins basically form a composite superfamily, which consists of five different families of exchanger proteins- YRBG, Na^+^/Ca^2+^ exchanger (NCX), Na^+^/Ca^2+^, K^+^ exchanger (NCKX), cation/Ca^2+^ exchanger (CCX), and H^+^/cation exchanger (CAX), which are classified on the basis of their function and evolutionary relationship (Cai and Lytton, 2004a; Emery et al., 2012; Pittman and Hirschi, 2016a). Recently, Singh et al. (2015) proposed the classification of all the CaCA superfamily proteins as NCX family due to the occurrence of Na_Ca_ex (PF01699) domain and tight evolutionary relationship between them. However, Pittman and Hirschi (2016a) by phylogenetic analysis and structure modeling further established that the CaCA superfamily members exhibit diverse structural and functional characteristics, and all of them cannot be classified as NCX proteins.

The CaCA superfamily proteins consist of comparable topological structure with an average of 10 transmembrane (TM) domains and two α-repeats region inside the TM 2-3 and 7-8. These repeats play vital role during ion-selectivity, binding and transportation by various groups of CaCA proteins (Kamiya and Maeshima, 2004; Ottolia et al., 2005; Shigaki et al., 2005; Nicoll et al., 2007). Recent crystallography analyses of certain CaCA proteins from archaea, bacteria and yeast provided detail insight into the structure (Liao et al., 2012; Nishizawa et al., 2013; Waight et al., 2013; Wu et al., 2013). The length of CaCA superfamily proteins varies from 300 to 1000 amino acid (AA) residues with almost similar topological structure. A typical CaCA protein consists of clusters of five TM helices in each half, separated by a cytosolic loop, with certain exceptions. For instance, the CCX proteins contain additional TM helix in C-terminal half (Cai and Lytton, 2004b). Further, the length of N-terminal region and cytosolic loop also varies in various groups of CaCA superfamily proteins (Emery et al., 2012).

Large scale genome analysis of numerous archaea, bacteria, fungi, algae, land plants, and animals established that- (1) the YBRG proteins are exclusively present in prokaryotes, (2) the CAX proteins are present in all organisms, (3) CCXs are present in all eukaryotes but not in prokaryotes, while (4) NCX and NCKX proteins are present in animal cells and algae, but are lacking in higher plants (Shigaki et al., 2006; Lytton, 2007; Emery et al., 2012; Khananshvili, 2013, 2014; Pittman and Hirschi, 2016a). However, land plants have evolved two additional groups of CaCA proteins- Mg^2+^/H^+^ exchanger (MHX) proteins and EF-hand domain containing CAX (EF-CAX) proteins (Emery et al., 2012; Gaash et al., 2013). The EF-CAX was also found in algae (Emery et al., 2012) and later named as NCX like protein (NCL) due to their functional divergence from CAX proteins (Wang et al., 2012; Li et al., 2016).

A plant MHX was first identified in *Arabidopsis thaliana* (AtMHX) as a member of NCX family, which enhanced Mg^2+^ concentration in vacuole without Na^+^/Ca^2+^ exchange activity (Shaul et al., 1999). Recently, the MHX proteins were identified from numerous land plants, which formed a distinct group with tight phylogenetic clustering (Emery et al., 2012; Gaash et al., 2013; Pittman and Hirschi, 2016a). The plant MHX proteins also differ from NCX proteins in certain key amino acid residues in α1 and α2 repeat regions responsible for Na^+^/Ca^2+^ exchange activity (Nicoll et al., 1996a; Iwamoto et al., 2000; Philipson and Nicoll, 2000; Ottolia et al., 2005; Emery et al., 2012). Furthermore, the MHX proteins consist of smaller central loop without Ca^2+^ binding domain (Lytton, 2007; Gaash et al., 2013).

The CAX proteins comprised the largest proportion of CaCA superfamily, and present in each group of organism (Shigaki et al., 2006; Manohar et al., 2011; Emery et al., 2012; Pittman and Hirschi, 2016b), except mammals and insects. These are broadly classified into type 1, type 2, and type 3 categories on the basis of phylogenetic clustering (Shigaki et al., 2006). Plants generally consist of type 1 CAX proteins, which are further divided into type 1A and type 1B groups (Shigaki et al., 2006; Emery et al., 2012). The CAX proteins usually exist as multi-gene families in higher plants. Generally, 5-6 CAX proteins are identified in various monocot and dicot plants, with a maximum of 14 CAX proteins in *Glycine max*, about half of them belonging to each type 1A and 1B groups (Emery et al., 2012; Pittman and Hirschi, 2016a). It has been suggested that both the groups differ in their ion selectivity; type 1B members like AtCAX2 and AtCAX5 facilitate the transportation of several ions such as Ca^2+^, Cd^2+^, and Mn^2+^, while type 1A proteins like AtCAX1 and AtCAX3 are thought to be specific for Ca^2+^ homeostasis (Hirschi et al., 2000; Shigaki et al., 2003; Edmond et al., 2009; Conn et al., 2011). However, *in-planta* and heterologous expression of type 1A CAX proteins in yeast facilitate transport of multiple ions (Kamiya et al., 2005; Shigaki et al., 2005, 2010; Koren'kov et al., 2007; Mei et al., 2009). Hence, CAX family proteins are recently referred as H^+^/cation exchangers.

The NCL proteins are reported as a new member of CaCA superfamily (Wang et al., 2012), separated from CAX family due to the presence of long cytoplasmic loop with EF-hand domains (Emery et al., 2012; Pittman and Hirschi, 2016a). These are identified in numerous land plants, and AtNCL is reported to perform Na^+^/Ca^2+^exchange activity (Emery et al., 2012; Li et al., 2016). However, tight phylogenetic clustering in conserved α2-repeat tree established that these proteins are evolutionary more closer to CAX proteins than NCX proteins (Emery et al., 2012; Pittman and Hirschi, 2016a).

The CCX family of proteins were identified from diverse groups of organisms including protozoa, invertebrate, and vertebrate animals, fungi and plants (Cai and Lytton, 2004a; Emery et al., 2012; Pittman and Hirschi, 2016a). Functional characterization revealed Na^+^(Li^+^)/Ca^2+^ exchanger activity in a mammalian CCX protein, earlier named as NCKX6 (Palty et al., 2004; Cai and Lytton, 2004b). However, CCX3 of Arabidopsis showed H^+^/K^+^ exchanger activity and it could also transport Na^+^ and Mn^2+^ but not Ca^2+^ (Morris et al., 2008). Phylogenetic analysis classified CCX proteins from various organisms into three subgroups. These results indicated functional variation in CCX family of proteins (Emery et al., 2012; Pittman and Hirschi, 2016a). Normally 3-6 CCX proteins are reported in land plants with a maximum of 8 in *Glycine max*. These are also found highly conserved across the organisms especially in α1 and α2 -repeat regions (Emery et al., 2012).

The CaCA superfamily proteins are functionally characterized in several plant species, which established their basic role in cation transport and homeostasis. Modulated expression of these proteins have also been observed in various plants during numerous abiotic stresses, which indicated their role in stress response (Hirschi et al., 1996, 2000; Shaul et al., 1999; Kamiya et al., 2005; Morris et al., 2008; Wang et al., 2012; Singh et al., 2015; Li et al., 2016). Functional characterization of certain CaCA proteins like AtNCL, AtCAX1, OsCAX4, SsCAX1 and others further established their role in various kinds of abiotic stress response (Mei et al., 2007; Han et al., 2012; Wang et al., 2012; Yamada et al., 2014; Li et al., 2016; Pittman and Hirschi, 2016b; Zhang et al., 2016).

Despite being very important proteins, CaCA superfamily has not been identified and characterized in *Triticum aestivum* (bread wheat), a staple food crop of about one-third population of world. *T. aestivum* consist of an allohexaploid genome (2n = 6x = 42), evolved by hybridization events of three sub-genomes A, B, and D (Marcussen et al., 2014). The unavailability of genomic information, composite nature of genome and complexity in application of modern functional genomics tools could be the probable reason for the same. In recent years, the genome sequence and numerous development and stress related high throughput RNA sequence data have been available from *T. aestivum* (IWGSC, 2014; Zhang et al., 2014b, 2016; Liu Z. et al., 2015; Pingault et al., 2015), which enabled the genome wide characterization of various gene families (Shumayla et al., 2016a,b; Zeng et al., 2016). Since the CaCA proteins are involved in several vital functions from development to stress response, there characterization becomes essential in an important food crop like *T. aestivum*.

Herein, we have performed the genome wide identification and comprehensive characterization of CaCA proteins (TaCaCA) in *T. aestivum* genome. They were further characterized for gene and protein structural organization, and phylogenetic relationship. Furthermore, the expression analysis of individual *TaCaCA* genes was carried out during various tissues developmental stages, and in biotic and abiotic stress conditions as well. The present study provided inclusive information about TaCaCA superfamily proteins that would be very useful in future crop development programs.

## Materials and methods

### Identification, classification and chromosomal distribution

To identify the CaCA superfamily proteins (TaCaCA) in the genome of *T. aestivum*, BLASTp search (*e*-value 0.00001) of known CaCA proteins sequences from rice and Arabidopsis (Emery et al., 2012; Gaash et al., 2013; Singh et al., 2015; Pittman and Hirschi, 2016a) was performed against the protein model sequences of *T. aestivum* obtained from IWGSC server (Nussbaumer et al., 2013; IWGSC, 2014) (http://www.wheatgenome.org/, http://wheat-urgi.versailles.inra.fr/Seq-Repository/Genes-annotations). A total of 6 CAX, 5 CCX and 1 each MHX and NCL sequences from Arabidopsis, and 6 CAX, 5 CCX, 2 MHX, and 2 NCL sequences from rice were used in BLAST search as described earlier (Pittman and Hirschi, 2016a). The putatively identified CaCA protein sequences were also searched against the newly reported protein model sequences (TGACv1) of *T. aestivum* generated by The Genome Analysis Centre (TGAC), available at Ensembl Plants (http://plants.ensembl.org/Triticum_aestivum/Info/Index) and other available sequences at NCBI database. The FGENESH pipeline (http://www.softberry.com/) was used for the prediction of coding sequence (Solovyev et al., 2006). The hydropathy analysis of putatively identified sequences was performed using TMHMMv2.01 (Krogh et al., 2001; Petersen et al., 2011) to further confirm their identity as CaCA proteins. The presence of EF-hand (PfamID: PF00036) and Na_Ca_ex (PfamID: PF01699) domains were confirmed by BLAST search against local pfam database (ftp://ftp.sanger.ac.uk/pub/databases/Pfam) following the method established in our laboratory (Finn et al., 2014; Shumayla et al., 2016a,b). CaCA superfamily proteins were also identified in *T. urartu* (TuCaCA) and *Aegilops tauschii* (AeCaCA) genomes, which are reported as progenitors of A and D subgenome of *T. aestivum*, respectively (Marcussen et al., 2014). The identified CaCA superfamily proteins were further classified into CAX, CCX, MHX, and NCL protein groups based on their relative sequence homology with known sequences from Arabidopsis and rice.

The chromosomal localization of *TaCaCA* genes was obtained by BLASTn search against available chromosome sequences of *T. aestivum* at URGI and Ensembl Plants (https://urgi.versailles.inra.fr/blast/, http://plants.ensembl.org/Triticum_aestivum/).

### Homeologs, orthologs and duplication events prediction

Bi-directional best hit approach at *e*-value 10^−10^ was used for the prediction of homeologous and orthologous genes as reported in earlier studies (Shumayla et al., 2016a,b). The identification of homeologous *TaCaCA* genes was based on their chromosome group, chromosome arm location and high percent (≥90%) identity. The identified homeologous genes were further confirmed by clustering them with *T. aestivum* unigene clusters (http://www.ncbi.nlm.nih.gov/UniGene). Further, the known pericentromeric inversion of chromosome 4AS-4AL (Liu et al., 1992) and chromosomal translocation 4AL-5AL (Hernandez et al., 2012) were also considered during homeolog identification. A unique name was assigned to each *TaCaCA* gene on the basis of their homeologous group and subgenome following the rules recommended for gene symbolization in *T. aestivum* (http://wheat.pw.usda.gov/ggpages/wgc/98/Intro.htm).

The orthologous proteins were identified by BLASTp search of TaCaCA protein sequences (*e*-value 10^−10^) against the protein model sequences of *T. urartu, Ae. tauschii*, rice and Arabidopsis downloaded from their respective databases (http://plants.ensembl.org, http://rice.plantbiology.msu.edu/, http://www.arabidopsis.org). Prediction of paralogous genes originated by duplication events were carried out by BLASTn search (at *e*-value 10^−10^) with high sequence similarity than the usual similarity between *TaCaCA* genes from different homeologous groups.

### Phylogenetic analysis

The phylogenetic analysis of TaCaCA superfamily proteins with various orthologs was performed using full-length, core domain and α2-repeat region sequences as reported in earlier studies (Emery et al., 2012; Pittman and Hirschi, 2016a). The core domain of individual proteins was obtained by removing the non-conserved N- and C-terminus portions, and central loop region as well. However, the conserved α2-repeat region in each protein was determined by aligning them with previously determined consensus sequence of CaCA proteins (Cai and Lytton, 2004a; Emery et al., 2012; Pittman and Hirschi, 2016a). The sequences were aligned using muscle program (Edgar, 2004) and phylogenetic tree was constructed by maximum likelihood method using MEGA7 with 1000 bootstrap replicates (Kumar et al., 2016).

### Sequence alignment and exon/intron organization

The multiple sequence alignments were carried out by using ClustalW (http://www.ebi.ac.uk/Tools/msa/clustalw2/), MAFFT (Katoh et al., 2002), muscle (Edgar, 2004), and Multalin (Corpet, 1988) programs. The genomic sequence of *T. aestivum* was downloaded from URGI (https://urgi.versailles.inra.fr/) and Ensembl Plants (http://plants.ensembl.org/Triticum_aestivum/) databases. The number of exons and introns in each *TaCaCA* gene were determined by aligning the coding sequences with genomic sequences. The number was further confirmed by blast search against *T. aestivum* genome dataset of IWGSC available at Ensembl Plants. The exon/ intron organization was analyzed using GSDS 2.0 (gsds.cbi.pku.edu.cn/) server (Hu et al., 2015).

### Protein characterization

The trans-membrane (TM) regions in individual TaCaCA protein was predicted using five different programs- TMHMM v2.0 (http://www.cbs.dtu.dk/services/TMHMM/), TMMOD, Phobius, SosuiG v.1.1 and DAS-Tmfilter (Hirokawa et al., 1998; Krogh et al., 2001; Cserzo et al., 2004; Käll et al., 2004; Kahsay et al., 2005; Petersen et al., 2011). The PROTTER v1.0 (http://wlab.ethz.ch/protter/start/) was also used for the prediction of TM topology (Omasits et al., 2014). The domain composition was established using Prosite-scan (http://prosite.expasy.org/scanprosite/), InterProScan (https://www.ebi.ac.uk/interpro/search/sequence-search), SMART (http://smart.embl-heidelberg.de/) and NCBI conserved domain blast (http://www.ncbi.nlm.nih.gov/Structure/bwrpsb/bwrpsb.cgi) servers (Jones et al., 2014; Letunic et al., 2014; Marchler et al., 2015). The domain architecture map was prepared using IBS (http://ibs.biocuckoo.org/online.php) server (Liu W. et al., 2015). The NCBI blast server (https://blast.ncbi.nlm.nih.gov/Blast.cgi) was used for similarity analysis with other sequences in the database. The localization of the protein within the cell was predicted using pSORT (http://wolfpsort.org/), CELLO v.2.5 (http://cello.life.nctu.edu.tw/), TargetP_v1, PredSL, ngLOC and ProtComp (http://www.softberry.com/berry.phtml) servers (Emanuelsson et al., 2000; Petsalaki et al., 2006; Yu et al., 2006; Horton et al., 2007; King et al., 2012). Occurrence of signal peptide was recognized using SignalP 4.1 (http://www.cbs.dtu.dk/services/SignalP/). The ExPasy compute pI/MW tool (http://web.expasy.org/compute_pi/) was used to compute the isoelectric point and molecular weight (Gasteiger et al., 2005).

### Expression analysis

The expression profile of *TaCaCA* genes was performed in various tissue developmental stages, and biotic and abiotic stress conditions using the high throughput RNA seq data. The tissue specific expression was analyzed using the data generated from three developmental stages of each root, leaf, stem, spike and grain in duplicates (Pingault et al., 2015). The data is available for download at https://urgi.versailles.inra.fr/files/RNASeqWheat/. The expression value was calculated as fragments per kilobase of transcript per million fragments mapped (FPKM) using RNA-Seq by Expectation-Maximization (RSEM) protocol from Trinity package (Li and Dewey, 2011; Haas et al., 2013). The tissue specific expression of individual gene was also confirmed at WheatExp server (http://wheat.pw.usda.gov/WheatExp/#) using the data (accession number: ERP004714) developed from similar tissues and developmental stages (Choulet et al., 2014).

The effect of biotic stress on the expression of *TaCaCA* genes was analyzed using the RNA seq data (accession number PRJNA243835) developed from leaves after 24 h of inoculation of fungus *Blumeria graminis f. sp. tritici* (Bgt) and *Puccinia striiformis* f. sp. *tritici* (Pst), separately (Zhang et al., 2014b). The RNA seq data was generated in triplicates and expression was calculated using Trinity package as described (Haas et al., 2013).

The effect of heat (HS), drought (DS), and combination of these stresses (HD) was analyzed using RNA seq data developed in duplicates (accession number SRP045409) from leaf samples after one and 6 h of incubation (Liu Z. et al., 2015). The effect of salt (NaCl) stress was studied using RNA seq data produced in three biological replicates (accession number SRP062745) from root tissues after 6, 12, 24, and 48 h of treatment (Zhang et al., 2016). The fold expression change of each *TaCaCA* gene under various stresses was also calculated in comparison to their respective controls using Trinity (Haas et al., 2013).

The genes having less than 10 FPKM expression value in each stage of an experiment were not included for evaluation or fold change calculations. The *TaCaCA* genes showing more than 2-fold up or down regulation were considered as differentially expressed. The Hierarchical Clustering Explorer 3.5 (http://www.cs.umd.edu/hcil/hce/) was used for the generation of heat-maps from various expression data.

## Results and discussion

### Identification, classification and genome wide distribution

A total of 34 TaCaCA superfamily proteins were identified in *T. aestivum* genome by extensive BLAST search of known sequences from rice and Arabidopsis (File S1). These were further confirmed by hydropathy analysis for the presence of TM domains, and conserved α1 and α2-repeat regions by aligning them with known sequences as suggested in earlier studies (Emery et al., 2012; Pittman and Hirschi, 2016a). The identified TaCaCA proteins were classified into 14 TaCAX, 10 TaCCX, 7 TaNCL, and 3 TaMHX proteins on the basis of their close similarity and tight phylogenetic clustering with respective known proteins from various plants (Figure S1; File S1). In other higher plants, a total of 14 (6 AtCAX, 5 AtCCX, 2 AtNCL, and 1 AtMHX), 15 (6 OsCAX, 5 OsCCX, 2 OsNCL, and 2 OsMHX), and 14 (6 ZmCAX, 6 ZmCCX, 1 ZmNCL, and 1 ZmMHX) CaCA protein are reported in Arabidopsis, rice, and maize, respectively (Emery et al., 2012; Singh et al., 2015; Pittman and Hirschi, 2016a). It was earlier established that *T. aestivum* consisted of higher number of genes in each gene family (Shumayla et al., 2016a,b) due to their large allohexaploid (2n = 6x = 42; AABBDD) nature of genome (Marcussen et al., 2014). Therefore, it was also expected in TaCaCA superfamily. Occurrence of 27 CaCA proteins (14 CAX, 8 CCX, 4 NCL, and 1 MHX) in a tetraploid soybean further established that the polyploid plants may contain more number of CaCA proteins than the diploid plants (Schmutz et al., 2010; Emery et al., 2012).

**Figure 1:**
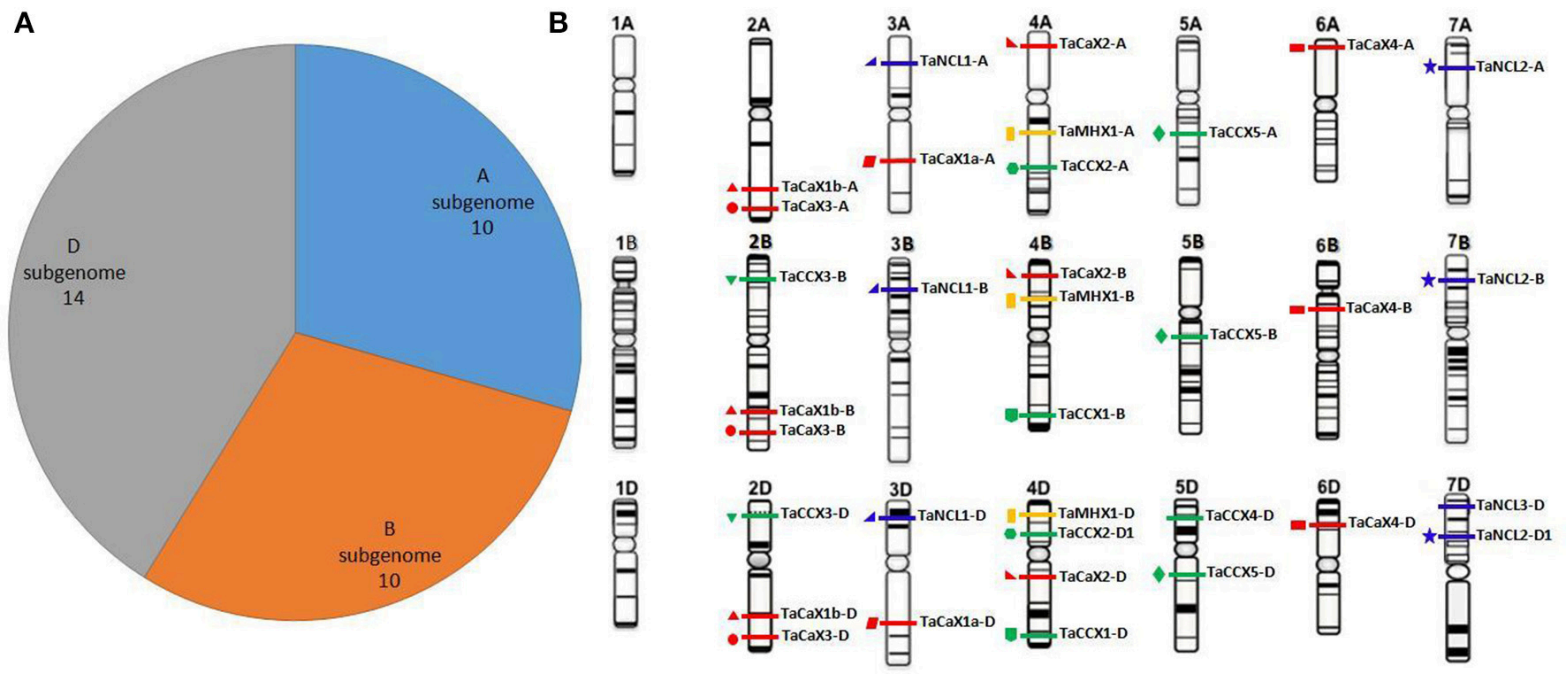
**Distribution of the ***TaCaCA*** superfamily genes on A, B, and D subgenomes and chromosomes**. Figure shows the frequency of *TaCaCA* genes on A, B, and D sub-genomes **(A)** and pictorial representation on various chromosomes **(B)**. The figures of various chromosomes are adapted from the IWGSC website (www.wheatgenome.org). The *TaCAX, TaCCX, TaNCL*, and *TaMHX* genes are represented by red, green, blue and yellow bars, respectively. The homeologous genes in each family are shown by similar symbol.

Genome-wide analysis established that the *TaCaCA* genes were derived from each subgenomes of *T. aestivum* as reported in case of other gene families (Shumayla et al., 2016a,b; Zeng et al., 2016). A total of 10 (5 TaCAX, 2 TaCCX, 2 TaNCL, and 1 TaMHX), 10 (4 TaCAX, 3 TaCCX, 2 TaNCL, and 1 TaMHX) and 14 (5 TaCAX, 5 TaCCX, 3 TaNCL, and 1 TaMHX) genes were located on A, B, and D subgenomes, respectively (Figure 1A). Since the A and D sub-genomes are derived from *T. urartu* and *Ae. tauschii* (Marcussen et al., 2014), we analyzed the occurrence of *CaCA* family genes in their recently reported genomic sequences (Jia et al., 2013; Ling et al., 2013) following the similar procedure. A total of 7 (3 TuCAX, 1 TuCCX, 2 TuNCL, and 1 TuMHX) and 11 (4 AeCAX, 4 AeCCX, 2 AeNCL, and 1 AeMHX) *CaCA* genes were found on *T. urartu* and *Ae. tauschii* genome, respectively (File S1), which was quite lower than genes detected on A and D sub-genomes of *T. aestivum*. The results indicated occurrence of duplication events during evolution of *TaCaCA* genes after hybridization of various sub-genomes. The detail about duplication is discussed in later section.

The *TaCaCA* genes were distributed on each chromosome group of A, B, and D sub-genome, except chromosome group 1 (Figure 1B). A maximum of four genes were located on chromosome 4D. In total four (chromosomes 2B, 2D, 4A, and 4B), five (chromosomes 2A, 3A, 3D, 5D, and 7D) and eight (chromosomes 3B, 5A, 5B, 6A, 6B, 6D, 7A, and 7B) chromosomes comprised three, two and one genes, respectively. Further analysis indicated that the various families of CaCA superfamily were basically located on a particular group of chromosome. For instance, the *TaCAXs* were found on chromosome groups 2, 3, 4, and 6; *TaCCXs* on chromosome groups 2, 4, and 5; *TaNCLs* on chromosome groups 3 and 7; while *TaMHXs* were located on chromosome group 4. Occurrence of CaCA genes on various chromosomes has also been reported in Arabidopsis and rice, where these genes are distributed on four out of five, and eight out of twelve chromosomes, respectively. Five genes of Arabidopsis are located on chromosome I, while three genes are reported on each of chromosome II, III and XI of rice (Singh et al., 2015).

### Prediction of homeologs and duplication events

Due to the composite allohexaploid (AABBDD) nature of genome, each progenitor diploid subgenome has contributed in the composition of various gene families in *T. aestivum* (Shumayla et al., 2016a,b; Zeng et al., 2016). Majority of genes are derived from homeologous chromosome groups from A, B, and D subgenomes, and those are considered as homeologous genes in various studies. These genes mostly share high (≥90%) sequence homology. The homeologous relation of *T. aestivum* genes has also been considered in gene symbolization and nomenclature (http://wheat.pw.usda.gov/ggpages/wgc/98/Intro.htm). We had also identified the homeologous *TaCaCA* gene on the basis of similarity. A total of 14 distinct clusters (5 *TaCAX, 5 TaCCX, 3 TaNCL*, and *1 TaMHX*) of homeologous genes were identified, majority of them consisted of at least one gene from each subgenome (File S2). However, a few homeologous genes were localized on either one or two subgenome. For instance, homeologous groups *TaCCX1* and *TaCCX3* were located on two (B and D) subgenomes, while *TaCCX4* and *TaNCL3* were found on only D subgenome (File S2). The number of distinct homeologous groups of *TaCaCA* genes was comparable to the total number of *CaCA* genes reported in diploid plant genomes like Arabidopsis and rice (Emery et al., 2012; Pittman and Hirschi, 2016a).

Gene duplication is earlier reported as a vital source for gene evolution (Magadum et al., 2013), which basically occurs due to retro-transpositions, imbalanced crossing over, and duplication of genome or chromosome. Occurrence of ~80% repeat sequences and various transposable elements in *T. aestivum* genome highly favors duplication as an important event during evolution (Marcussen et al., 2014; Glover et al., 2015). Several duplication events (DEs) are earlier predicted in various gene families of *T. aestivum* on the basis of high (≥80%) similarity (Shumayla et al., 2016a,b), but it was difficult in case of *TaCaCA* superfamily genes because of high divergence among various families of genes (File S3). The genes found on same subgenome and showed relatively higher similarity than the other genes were considered as putatively duplicated genes. Further, the information about *CaCA* genes was merely available from A (*T. urartu*) and D (*Ae. tauschii*) subgenome progenitors, therefore the DEs in *TaCaCA* genes were only predicted on these subgenomes.

The A subgenome of *T. aestivum* consisted of five *TaCAX* and two *TaCCX* genes, while their progenitor *T. urartu* had three *TuCAX* and one *TuCCX*. This indicated occurrence of at least two and one DEs during evolution of *TaCAX* and *TaCCX* genes, respectively. Probably, *TaCAX3-D* and *TaCAX4-D* were evolved by putative duplication of *TaCAX2-D* (File S3). In case of *TaCCX*, though both the genes (*TaCCX2-A* and *TaCCX5-A*) shared less than 50% sequence similarity, still these might be putative paralogous genes. The chromosomal translocation event between 4AL-5AL (Hernandez et al., 2012) also supports the same. Similarly, one DE was predicted in each *TaCAX, TaCCX*, and *TaNCL* gene family on D subgenome, because the progenitor *Ae. tauschii* was short of one gene in these families. The gene pairs *TaCAX1a-D* and *TaCAX1b-D, TaCCX2-D* and *TaCCX4-D, TaNCL1-D* and *TaNCL3-D* could be predicted as putative duplicated genes in D subgenome. Role of duplication events in evolvement of *CaCA* superfamily genes has also been earlier reported from both plant and animal species (On et al., 2008; Emery et al., 2012).

### Phylogenetic analysis

**Figure 2:**
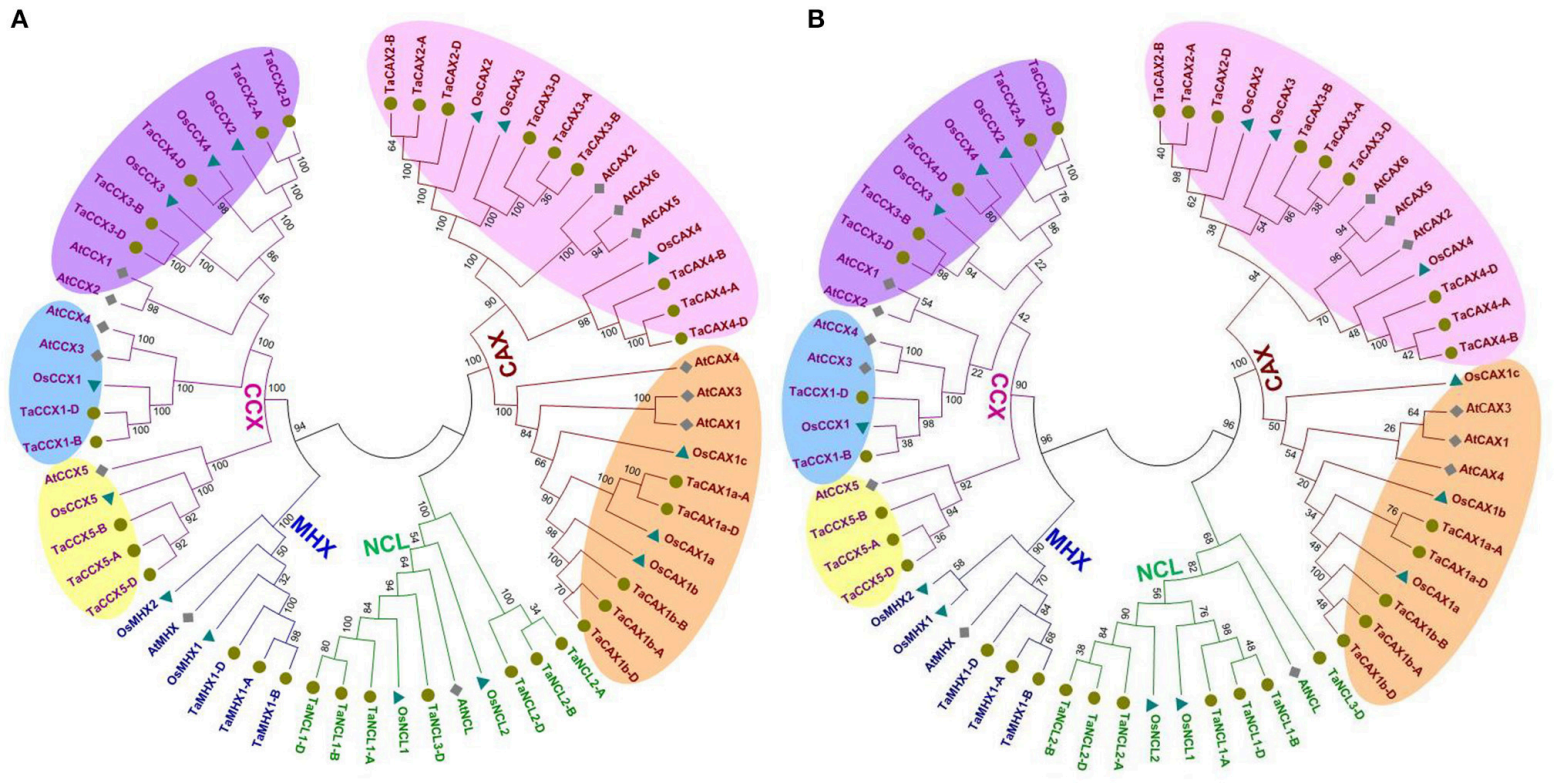
**Phylogenetic analysis of the CaCA superfamily proteins**. The evolutionary relationship between CaCA proteins of *T. aestivum*, Arabidopsis, and rice was analyzed using conserved core domain **(A)** and α2-repeat motif **(B)**. Figure shows tight clustering of the CAX, CCX, NCL, and MHX family proteins from various plants, which are shown with red, purple, green, and blue color lines and fonts, respectively. The CAX and CCX family proteins are further classified into two (Type 1A and Type 1B) and three (group 1, 2, and 3) categories, respectively. Orange and pink shaded regions show Type 1A and Type 1B CAX proteins, while yellow, blue and purple shaded regions represent group 1, 2, and 3 CCX proteins, respectively.

In earlier studies, usually three phylogenetic trees have been constructed using full length, core domain and α2-repeat motif sequences to get better separation of various members of CaCA superfamily proteins. It is due to the poor alignment of full length CaCA protein sequences especially in N- and C-terminus regions, variation in length of cytoplasmic loop and lack of α1-repeat motif in NCL proteins (Emery et al., 2012; Wang et al., 2012; Pittman and Hirschi, 2016a). Similar phylogenetic trees were constructed in the present study using CaCA protein sequences from various plant species. Four distinct clades of CAX, CCX, NCL, and MHX family proteins were found in each tree, which clearly depicted high homology within each family, and evident distinction between different families (Figure 2, Figure S1). Though, the NCL and MHX proteins formed distinct clades but showed tight phylogenetic relation with CAX and CCX proteins, respectively. Similar clustering is reported in earlier studies (Emery et al., 2012; Pittman and Hirschi, 2016a).

The identified homeologous TaCaCA proteins from A, B, and D subgenomes of *T. aestivum* were closely clustered due to the high homology between them. The CaCA proteins identified from *T. urartu* and *Ae. tauschii* genomes were located in proximity to the related proteins from A and D subgenome of *T. aestivum*, respectively (Figure S1). It was probably due to their origin from respective subgenomes (Marcussen et al., 2014). For instance, TaNCL2-A and TaNCL2-D were proximally clustered with TuNCL2 and AeNCL2, respectively. Most of the predicted orthologous sequences from Arabidopsis and rice were clustered together with their respective TaCaCA proteins in various clades, such as CCX5 proteins from Arabidopsis, rice and *T. aestivum* genome were grouped together (Figure 2A). The evolutionary relation between orthologous CaCA proteins across the various plant species has also been observed earlier (Emery et al., 2012; Pittman and Hirschi, 2016a). Since the land plants are evolved from a common ancestor (McClung, 2010), they are found to be evolutionary related in the form of orthologous genes. The orthology of various other gene families of *T. aestivum* has also been earlier established with several land plants (Zeng et al., 2016; Shumayla et al., 2016a,b).

Since the CAX and CCX proteins are further classified into various groups on the basis of significant variation in their sequences and functional divergence (Cai and Lytton, 2004b; Palty et al., 2004; Shigaki et al., 2006; Morris et al., 2008; Pittman et al., 2009; Manohar et al., 2010; Emery et al., 2012), the phylogenetic trees developed from core domain and α2-repeat motif were also analyzed in terms of separation of those groups (Figures 2A,B). The OsCCX5 sequence did not contain α2-repeat motif, therefore it was not included in α2-repeat motif phylogeny. In plants, CAX proteins are basically divided into Type1A and Type1B groups, while CCXs comprised group 1, 2 and 3 (Emery et al., 2012). Type1A and Type1B CAX groups were clearly separated in both the trees, which indicated evolutionary divergence in TaCAX proteins, as earlier reported in other plants (Emery et al., 2012). In case of CCX, the phylogenetic tree developed from core domain sequences showed distinct separation of group 1, 2, and 3 proteins than the α2-repeat motif phylogeny (Figures 2A,B). For instance, AtCCX1 and AtCCX2 were clustered with group 3 CCX proteins in core domain phylogeny (Figure 2B) as reported in earlier study (Emery et al., 2012), while they were found closer to group 2 in α2-repeat motif phylogeny (Figure 2A). It is probably due to the conserved nature of this motif.

### Gene and protein characterization

**Figure 3:**
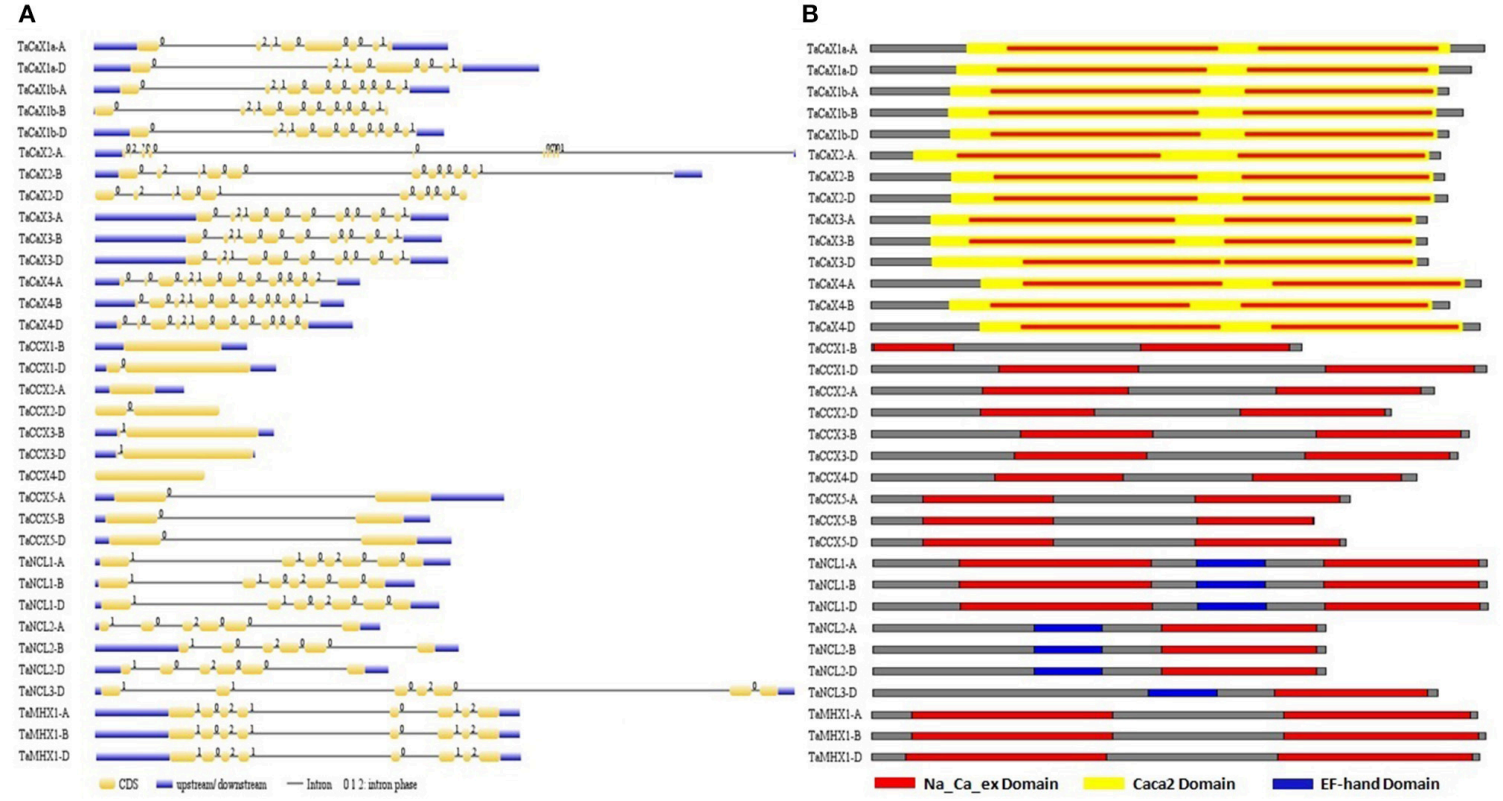
**Gene and protein structure analysis. (A)** Intron/exon configuration of *TaCaCA* superfamily genes. Exons and introns are shown as yellow boxes and black lines, respectively. Un-translated regions (UTRs) are shown as blue boxes. Various intron phases are represented as- 0; intron phase 0, 1; intron phase 1, 2; intron phase 2. **(B)** Figure shows domain architecture of TaCaCA proteins. The red, yellow and blue colored boxes represent Na_Ca_ex, Caca2 and EF-hand domains, respectively.

Several standard methods are reported for the characterization and comparison of different features of various genes in a gene family (Singh et al., 2015; Shumayla et al., 2016a,b; Zhou et al., 2016). The *TaCaCA* superfamily genes and proteins were analyzed in terms of various characteristic features like exon/intron organization, intron phase, length, molecular weight, pI, transmembrane, cytoplasmic loop size, cellular localization and domain architecture (Figure 3; Table 1; Files S4–S8). Out of 34 *TaCaCA* genes, 31 consisted of one or more introns, while three genes (*TaCCX1-B, TaCCX2-A*, and *TaCCX4-D*) were intron less. The average number of introns present in *TaCAX, TaCCX, TaNCL*, and *TaMHX* gene families were 10, 0.8, 5.5, and 7, respectively. A maximum of 12 introns were found in each *TaCAX4-A* and *TaCAX4-D* gene. The identified homeologus genes consisted of almost similar pattern of exon/ intron distribution (Figure 3A; Table 1; Files S4). The analysis of intronic phase indicated occurrence of 62, 23 and 14% introns in 0, 1, and 2 phase, respectively (Figure 3A). Similar distribution of these intron phases has been earlier reported in various organisms, which suggested the conserved nature of evolution of eukaryotic gene structure (Fedorov et al., 1992).

**Table 1:** **General characteristic features of TaCaCA superfamily proteins**.

		**TaCAXs**	**TaCCXs**	**TaNCLs**	**TaMHXs**
Protein length (AAs)	Maximum	462	636	580	524
	Minimum	419	445	427	517
	Average	438.3	543.2	507.4	520
MW (kDA)	Maximum	49.1	68.3	63.1	58.2
	Minimum	45.7	47.9	47.3	57.5
	Average	47.3	57.4	55.7	57.8
pI	Maximum	6.21	8.84	5.97	5.17
	Minimum	4.85	5.06	5.3	5.08
	Average	5.31	7.71	5.5	5.13
Transmembrane	Maximum	11	15	12	11
	Minimum	8	9	6	7
	Average	10.17	11.7	9.03	9.2
Loop size	Maximum	56	123	164	118
	Minimum	23	69	161	118
	Average	33.4	86.1	163.5	118
Intron	Maximum	12	2	6	7
	Minimum	7	0	5	7
	Average	10	0.8	5.5	7

The average length of TaCAX, TaCCX, TaNCL, and TaMHX family proteins were 438, 543, 507, and 520 AA residues, respectively. However, the average molecular weight (MW) and pI of these families were 47, 57, 55, and 57 kDa, and 5.3, 7.7, 5.5, and 5.1, respectively (Table 1; File S4). The TaCCX1-D and TaCAX3-A with MW 68.3 and 45.7 kDa were the biggest and smallest TaCaCA proteins. Variation in length and size of various CaCA superfamily proteins has also been observed in earlier studies. The AtCCX4 (AT1G54115; 645 AAs) and OsCCX1 (LOC_Os03g08230; 639 AAs) are the largest, while AtCCX5 (AT1G08960; 416 AAs) and OsCCX5 (LOC_Os11g05070; 265 AAs) are the smallest CaCA proteins reported from Arabidopsis and rice, respectively (Emery et al., 2012; Singh et al., 2015).

Since the CaCA proteins are membrane bound in nature (Cai and Lytton, 2004a; Emery et al., 2012), the occurrence of TM regions and sub-cellular localization was also predicted using various tools. Further, the number of TMs predicted by various tools was varied in majority of proteins as reported in earlier studies (Emery et al., 2012; Pittman and Hirschi, 2016a), therefore the average occurrence was calculated (File S5). The average number of TM regions found in TaCAX, TaCCX, TaNCL, and TaMHX family proteins was 10, 11, 9, and 9, respectively. However, the average TMs in total TaCaCA proteins was 10 (Table 1; File S5). Similar occurrence of TMs is reported in various other plants species (Emery et al., 2012), which supported the accuracy of our finding. As reported in other organisms (Cai and Lytton, 2004a,b; Emery et al., 2012), the TM regions were found in two groups in N- and C-terminus half of each TaCaCA protein, which were separated by a cytoplamic loop (CL) (Figures S2–S6). The average N- and C-terminus TMs ratio was 6:5, 5:6, 6:5, and 5:5 in TaCAX, TaCCX, TaNCL, and TaMHX family proteins, respectively. The length of CL also varied in various families due to the presence and absence of their respective domains in this region (File S6). The TaNCL proteins consisted of longest CL (~163 AAs), which is due to the presence of EF-hand domain (Figure S4, File S8). The average size of CL in TaCAX, TaCCX, and TaMHX proteins was ~33, ~86 and ~118 AAs, respectively. About similar length of CL is reported in other plant species (Emery et al., 2012).

The sub-cellular localization analysis indicated large discrepancy in prediction of various tools (File S7). The CELLO server predicted localization of each protein in plasma membrane, while ProtComp predicted most of them as vacuolar. However, other tools showed assorted results. The incongruity in prediction of these servers has also been reported in earlier studies (Vaid et al., 2012). This might be due to the differences in the algorithm of prediction of each tool. To validate the prediction, we analyzed the localization known proteins like AtCAX1, AtCAX2, and AtMHX, which are established as vacuolar proteins (Shaul et al., 1999; Hirschi et al., 2000; Kamiya et al., 2006). We found ProtComp prediction was quite reasonable, which predicted vacuolar localization for the majority of proteins except TaCCX5 homeologs. However, it could not provide localization of NCLs. The occurrence of CaCA proteins in plasma membrane and vacuole has been earlier reported (Wang et al., 1994, 2012; Bickerton and Pittman, 2015; Singh et al., 2015), but the actual localization of each TaCaCA superfamily protein need to be established.

**Figure 4:**
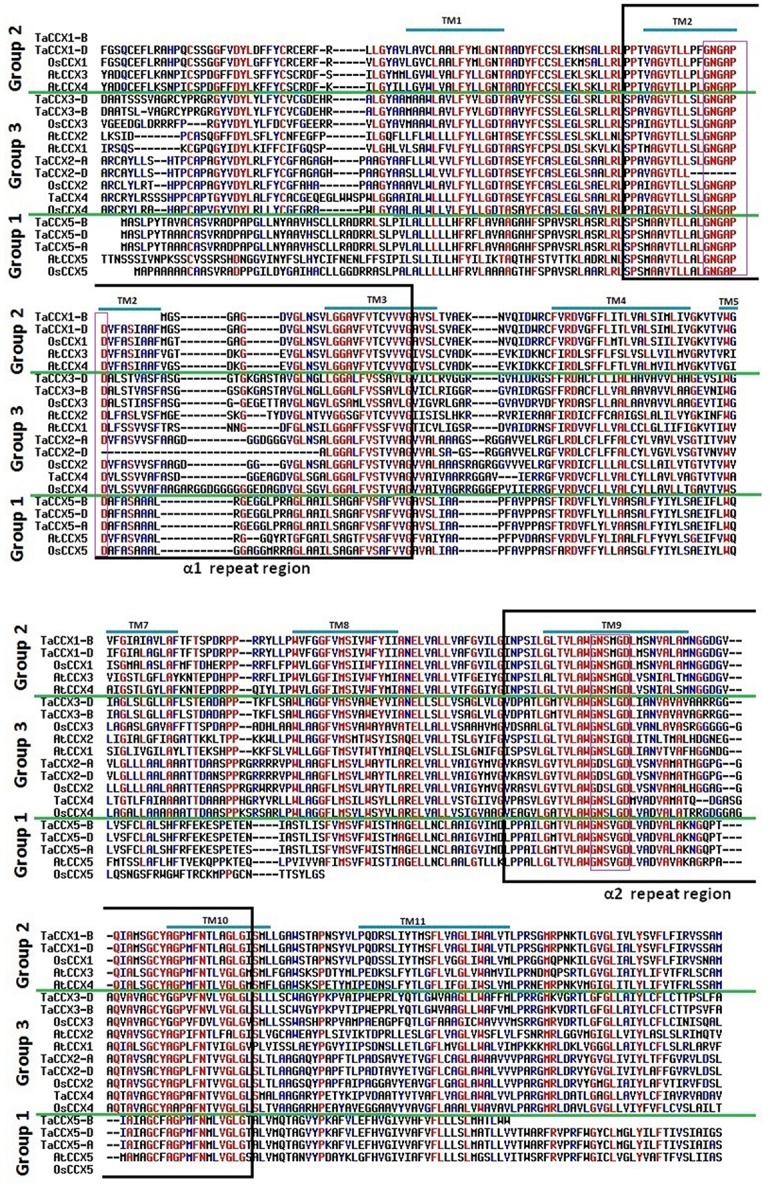
**Multiple sequence alignment of CCX proteins**. Figure shows alignment of CCX protein sequences of *T. aestivum*, Arabidopsis and rice. The group 1, 2, and 3 CCX sequences are separated by green lines. Conserved α1 and α2-repeat regions are shown in black colored boxes. The signature motifs “GNG(A/S)PD” in α1-repeat and “G(N/D)SxGD” in α2-repeat motif reported by Cai and Lytton (2004a) are shown in purple boxes. The predicted transmembrane (TM) spans are over-lined in blue color.

**Figure 5:**
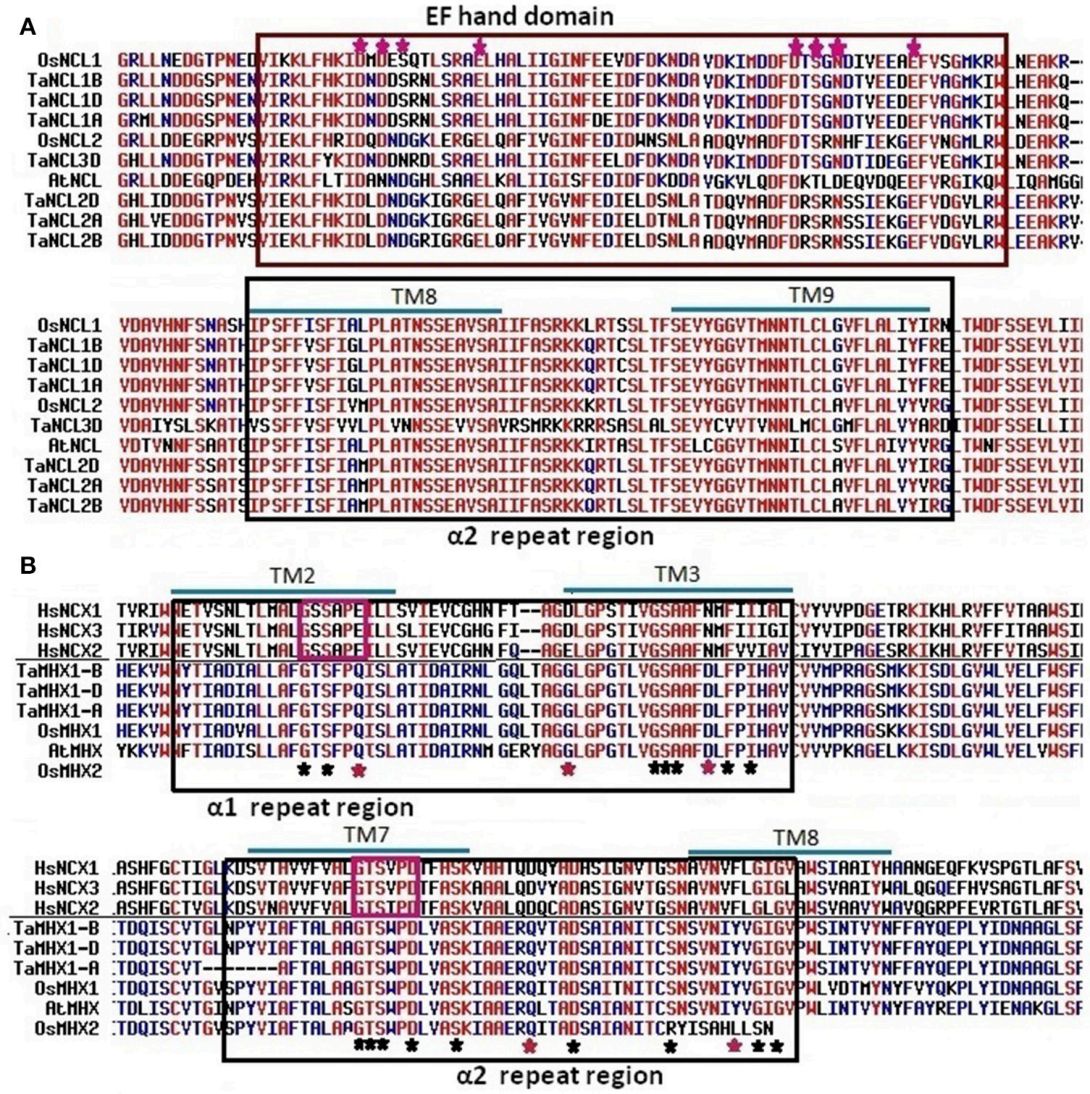
**Multiple sequence alignments of NCL and MHX proteins. (A)** Figure shows alignment of EF-hand domain and α-2 repeat region of NCL proteins from *T. aestivum*, Arabidopsis, and rice. Pink asterisks indicate calcium binding sites in EF-hand domain. **(B)** Alignment of α-1 and α-2 repeat regions of human NCXs (HsNCX1; NP_066920, HsNCX2; NP_055878 and HsNCX3; NP_891977) with MHX proteins from *T. aestivum*, Arabidopsis and rice. The signature motifs “GSSAPE” in α1-repeat and “GTSVPD” α2-repeat of human NCX protein are shown in pink coloured boxes. Residues involved in Na^+^/Ca^2+^ exchange in HsNCX1 are indicated by asterisks, while the red asterisks indicates the residues which are not conserved in the MHX proteins.

The domain composition and their organization play vital role in determining the fundamental function of a protein. Numerous *in-silico* methods and databases like SMART (Letunic et al., 2014), Scan-Prosite, InterProScan (Jones et al., 2014), NCBI-Conserved Domain Database (Marchler et al., 2015) are available to analyse the domain architecture of proteins. These have been used for the domain analysis of a variety of proteins including CaCAs from various plants and animal species (Upadhyay et al., 2013, 2016; Singh et al., 2015; Shumayla et al., 2016a,b). Each TaCaCA protein consisted of one or more Na_Ca_ex domain (PF01699) (Figure 3B; File S8), as reported in case of other plants (Singh et al., 2015). All the TaCAX, TaCCX, TaMHX, and TaNCL1 group proteins comprised two Na_Ca_ex domains, one in each N- and C terminus half (Figures 4–5; File S8; Figures S2–S6). However, the TaNCL2 and TaNCL3 proteins consisted of a single Na_Ca_ex domain, which was located in C-terminus half. Additionally, TaCAXs and TaNCLs consisted of caca2 (TIGR00846) and EF-hand (PF00036) domain, respectively. The caca2 domain was basically located throughout the core domain of each TaCAX protein, whereas the Ef-hand domain was positioned in CL region of TaNCLs (Figure 5B; File S8). Since each CaCA superfamily protein consisted of Na_Ca_ex domain, Singh et al. (2015) proposed their nomenclature as NCX proteins instead of classifying them in several families. However, the various families of CaCA superfamily differ significantly in their structure, conserved motifs and function (Cai and Lytton, 2004a; Emery et al., 2012). Further, these formed distinct phylogenetic groups in each organism, which suggested that their classification in different families is more meaningful rather than naming all of them as NCX proteins (Pittman and Hirschi, 2016a).

Since the numerous conserved motifs and AAs play significant role in functional divergence of various families of CaCA proteins (Cai and Lytton, 2004a; Shigaki et al., 2006; Emery et al., 2012; Gaash et al., 2013), their occurrence and conservation was also analyzed in TaCaCAs by multiple sequence alignments with the known proteins. As reported in other plant species, all the TaCAX proteins consisted of both the α-repeat motifs and various other conserved AA residues. The signature motif “GNxxE” was also found conserved in α-repeats of TaCAX proteins, which was followed by a conserved H and A residue in the α2-repeat region (Figure S2). Both the acidic E residues are reported as essential for ion transport. The conserved H residue is also found important, which could not be exchanged with R or A residue (Kamiya and Maeshima, 2004). Furthermore, we observed “GGLLNAT” and SLLGS(I/V)LSN(L/M)LLV(L/V)G in α1-repeat, and “FIS(I/V)IL(L/I)PIV” in α2-repeat as highly conserved motif in TaCAX protein along with the CAXs of Arabidopsis and rice. Besides above motifs, numerous conserved G residues were also found, which may provide conformational flexibility to the CAX proteins as reported in earlier studies (Yan and Sun, 1997; Shigaki et al., 2006). The TaCAX proteins also formed two distinct groups, which could be easily marked as Type 1A and Type 1B on the basis of their similarity with the known proteins from other plant species (Figure S2). Although, the α-repeats were quite conserved, but both group of proteins differ significantly in AAs composition especially between TM-4 and TM-8, and beyond TM-9. The Type 1A CAX appeared to be more hydrophilic along with the numerous acidic residues like E and D. Similar observation is earlier reported in other plants (Shigaki et al., 2006; Emery et al., 2012), however, role of these conserved motifs and residues needs to be established in future studies.

Similar to the CCX proteins in other organisms (Cai and Lytton, 2004a; Emery et al., 2012), the TaCCXs also comprised two α-repeat motifs around TM2-TM3 and TM9-TM10 (Figure 4; Figure S3). The signature motifs “GNG(A/S)PD” in α1-repeat and “G(N/D)SxGD” in α2-repeat were too found conserved in TaCCX proteins except TaCCX2-D, which lacked former one. Further, motifs “A(G/A)VTLL” and “LGxTVLAW” were highly conserved in proximity to the signature motifs in α1 and α2-repeats, respectively. The TaCCX proteins could be classified into three groups on the basis of their sequence similarity with known CCX proteins of rice and Arabidopsis. Despite of high similarity in α-repeat regions, the CCX sequences from various groups showed significant divergence, which may also be responsible for functional diversification as reported in earlier studies (Cai and Lytton, 2004a; Palty et al., 2004; Morris et al., 2008; Emery et al., 2012). Moreover, functional characterization of each group of CCX protein needs to be individually established especially for their substrate specificity.

Despite of similar 6:5 topology of TM domains, the NCL proteins are separated from CAXs due to the high dissimilarity in their sequences (Emery et al., 2012). Sequence alignment showed significant variation between TaCAX and TaNCL family proteins (Figure S5). The NCL proteins including TaNCLs merely comprised α2-repeat motif and lacked α1-repeat (Figure 5A; Figure S4-S5). It has been earlier demonstrated that the occurrence of two α-repeats are necessary to form a functional protein (Nicoll et al., 2007). The separated N- and C-terminus portions of AtCAX1 could not perform transportation (Zhao et al., 2009), but the mutated NCKX6 with single α-repeat motif appeared functional after the oligomerization of protein (Palty et al., 2006). Moreover, AtNCL is established as functional protein, and it is suggested that certain critical AA residues like E or D from α1 and α2-repeats, S or T from extracellular Na^+^ binding site, and T, S and N from intracellular Na^+^ region are conserved in this protein (Wang et al., 2012). However, the detailed functional and structural characterization of individual NCL proteins is still required. The NCL proteins varied from CAXs in signature sequence of α2-repeat motif as well (Figure S5). In contrary to the NCX proteins (Nicoll et al., 1990, 1996a,b), the TaNCL proteins lacked long (~230 AAs) calcium binding domain (CBD) in CL regions (Figure S6), but consisted a small (~65 AAs) EF-hand domain with calcium binding motifs (CBMs) (Figure 5A). The CBMs mostly comprised acidic and/or negatively charged amino acid residues such D, E, S, and N, which suggested their electrostatic interaction with positively charged Ca^2+^ ions. The binding of Ca^2+^ ions persuade conformational shifting in EF-hand domain, which is responsible for activation and inactivation of the target proteins (Ikura, 1996). The CBMs of EF-hand domain were also found conserved in NCL proteins reported from other plant species (Emery et al., 2012).

The MHX proteins are recently reported to be evolved as new family within the CaCA superfamily (Gaash et al., 2013). These are exclusively reported to be present in plants, which are probably evolved from NCX proteins after separation of the streptophytes and chlorophytes (Yoon et al., 2004; Gaash et al., 2013). Similar to the NCX proteins, MHXs also consisted of two α-repeats, but differed in signature motifs and lacked long cytoplasmic Ca^2+^ binding domain (Figure 5B, Figure S7). The MHX proteins comprised signature motifs “GTSFPQ” and “GTSWPD” in place of “GSSAPE” and “GTSVPD” in NCX at α1 and α2-repeats, respectively. Some of the key residues like E and D in α1-repeat, and D and F in α2-repeats are substituted with other AA residues in the MHXs (Emery et al., 2012; Gaash et al., 2013). Similar changes had been marked in case of TaMHXs. These residues play vital role in Ca^2+^ ion transport in NCX (Nicoll et al., 1996a). Mutation in these regions substantially reduced their affinity (Iwamoto et al., 2000), which explained the lack of Na^+^/Ca^2+^ exchange activity by MHX proteins. Moreover, the MHX transporters also lacked the Ca^2+^ binding domain in central loop region (Lytton, 2007). Certain other motifs like “ASKIA,” “TADSAI” and “ELGGP” reported as conserved in plant MHXs (Gaash et al., 2013) could also be found in TaMHXs (Figure S7). Since the AA residues in α-repeat regions play important role in ion selectivity (Shigekawa et al., 2002; Ottolia et al., 2005), sequence variation in these might be responsible for diversification in ion selectivity of various CaCA proteins. AtMHX is earlier reported to be involved in vacuolar transportation of Mg^2+^, Zn^2+^, Cd^2+^, and probably Fe^2+^ against protons (Shaul et al., 1999; Berezin et al., 2008). Since the plant MHXs share comparable structural organization with significant conservation, they may also be involved in analogous functions, but that needs to be established in individual proteins.

### Expression analysis in tissue developmental stages

**Figure 6:**
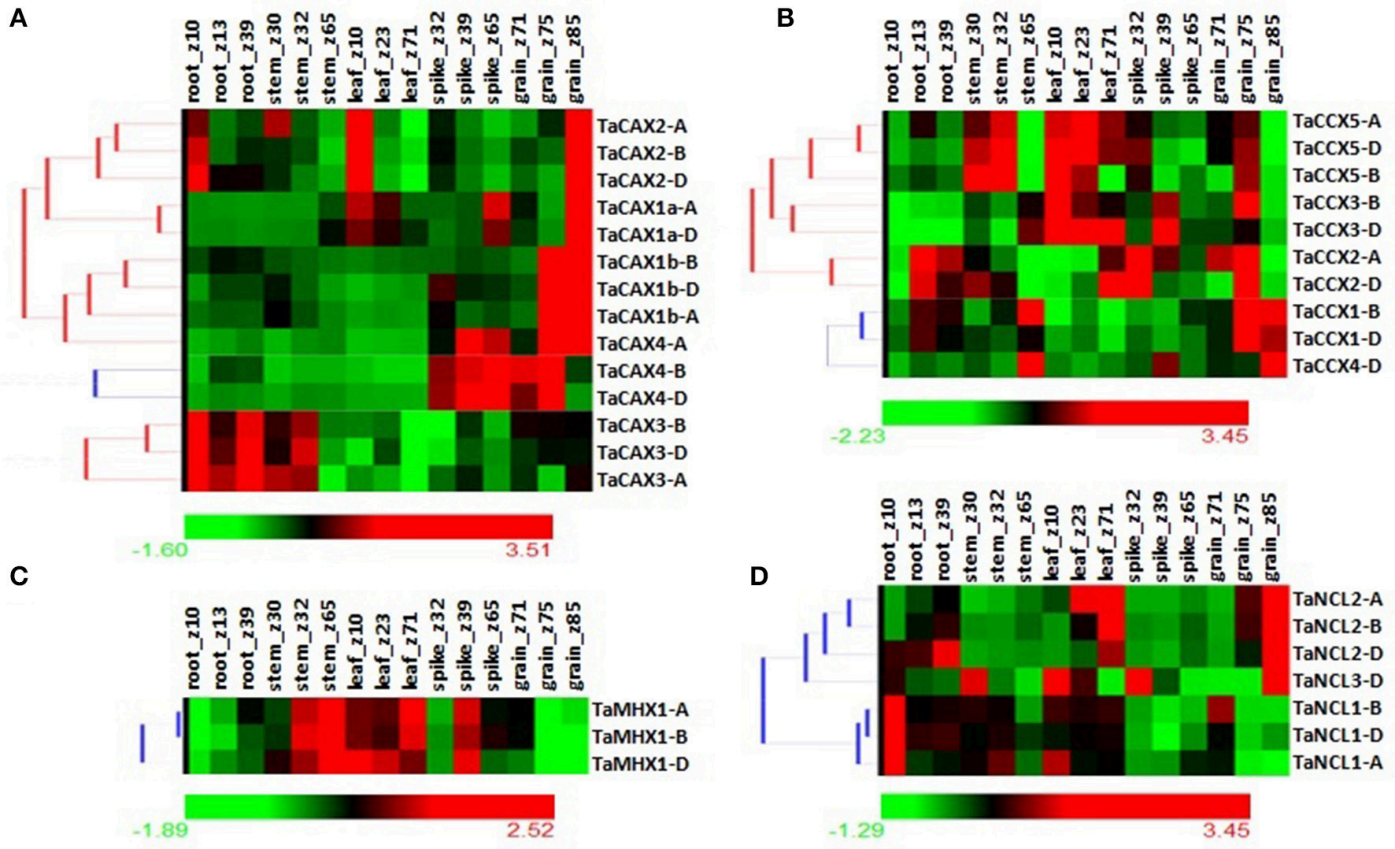
**Relative expression profile of ***TaCaCA*** superfamily genes in various tissue developmental stages**. Heat map shows relative expression profile of **(A)** *TaCAX*, **(B)** *TaCCX*, **(C)** *TaMHX*, and **(D)** *TaNCL* genes in three developmental stages of each root, leaf, stem, spike, and grain tissue. The developmental stages are shown in Zadoks scale.

Calcium ion is an essential secondary messenger in plants which play vital roles in various developmental, biochemical, and physiological processes. The expression analysis is a method to predict the putative function of an unknown gene (Zeng et al., 2016). Further, *CaCA* superfamily genes are also reported to play vital role in plant development (Shaul et al., 1999; Cheng et al., 2005; Wang et al., 2012; Pittman and Hirschi, 2016b). Therefore, their expression was analyzed in various developmental stages of *T. aestivum* using high throughput transcriptome data (Pingault et al., 2015). Each *TaCaCA* gene showed significant but varied expression in different developmental stages of all the analyzed tissues. Expression of *CAX* genes was usually high in each developmental stage as compared to the other genes. Likewise high expression of CAX genes in different tissues has also been reported in Arabidopsis and rice (Cheng et al., 2002, 2005; Pittman et al., 2004; Kamiya et al., 2005, 2006, 2012; Mei et al., 2009; Wang et al., 2012; Bickerton and Pittman, 2015; Singh et al., 2015). The *TaCAX1a-A, TaCAX1a-D, TaCAX1b-A, TaCAX1b-B*, and *TaCAX1b-D* in *CAX* family, TaCCX1*-B, TaCCX1-D, TaCCX2-A* and *TaCCX2-D* in *CCX* family, *TaNCL1-A, TaNCL1-B*, and *TaNCL1-D* in *NCL* family, and *TaMHX1-D* in *MHX* family were relatively high expressing genes in one or more developmental stages (Figure 6; File S9). The majority of *TaCAX* genes were highly expressed in grain except *TaCAX3* group genes, which were highly expressed in root and stem (Figure 6A). The high expression of *CAX* genes is also reported in seed of rice and Arabidopsis (Goel et al., 2012; Bickerton and Pittman, 2015).

In case of *CCX* family genes, *TaCCX1* and *TaCCX4* were highly expressed in grain, *TaCCX5* in leaf and stem, *TaCCX3* in leaf and grain, while *TaCCX2* in root, spike and grain (Figure 6B). The orthologous *CCX* genes from rice and Arabidopsis also showed comparable expression pattern (Morris et al., 2008; Singh et al., 2015). The *TaMHX* genes were highly expressed in stem, leaf and spike up to certain extent (Figure 6C) as observed in case of other plants (Singh et al., 2015). Since MHX transporter is usually involved in Mg^2+^ transportation (Shaul et al., 1999), which plays vital role in chlorophyll synthesis, this might be the reason for their high expression in green tissues. Furthermore, we could not observe significant expression of *TaMHX* in grain and root developmental stages. The over expression of AtMHX caused reduced growth in transgenic tobacco and Arabidopsis plants, which indicated their role in development (Berezin et al., 2008). An interesting expression pattern of *TaNCL* genes was observed. The *TaNCL1* group genes were high expressing in root, while *TaNCL2* in grain and leaf. *TaNCL3* showed assorted expression in various tissues (Figure 6D). Comparable expression of these genes is reported in other plants also (Singh et al., 2015). The variable expression of *TaCaCA* genes in different tissue developmental stages indicated their specific role during development. However, constitutively high expression of certain genes like *TaCAX1-A* and *TaNCL1-B* suggested their role throughout the various tissue developments.

### Expression analysis in biotic and abiotic stresses

**Figure 7:**
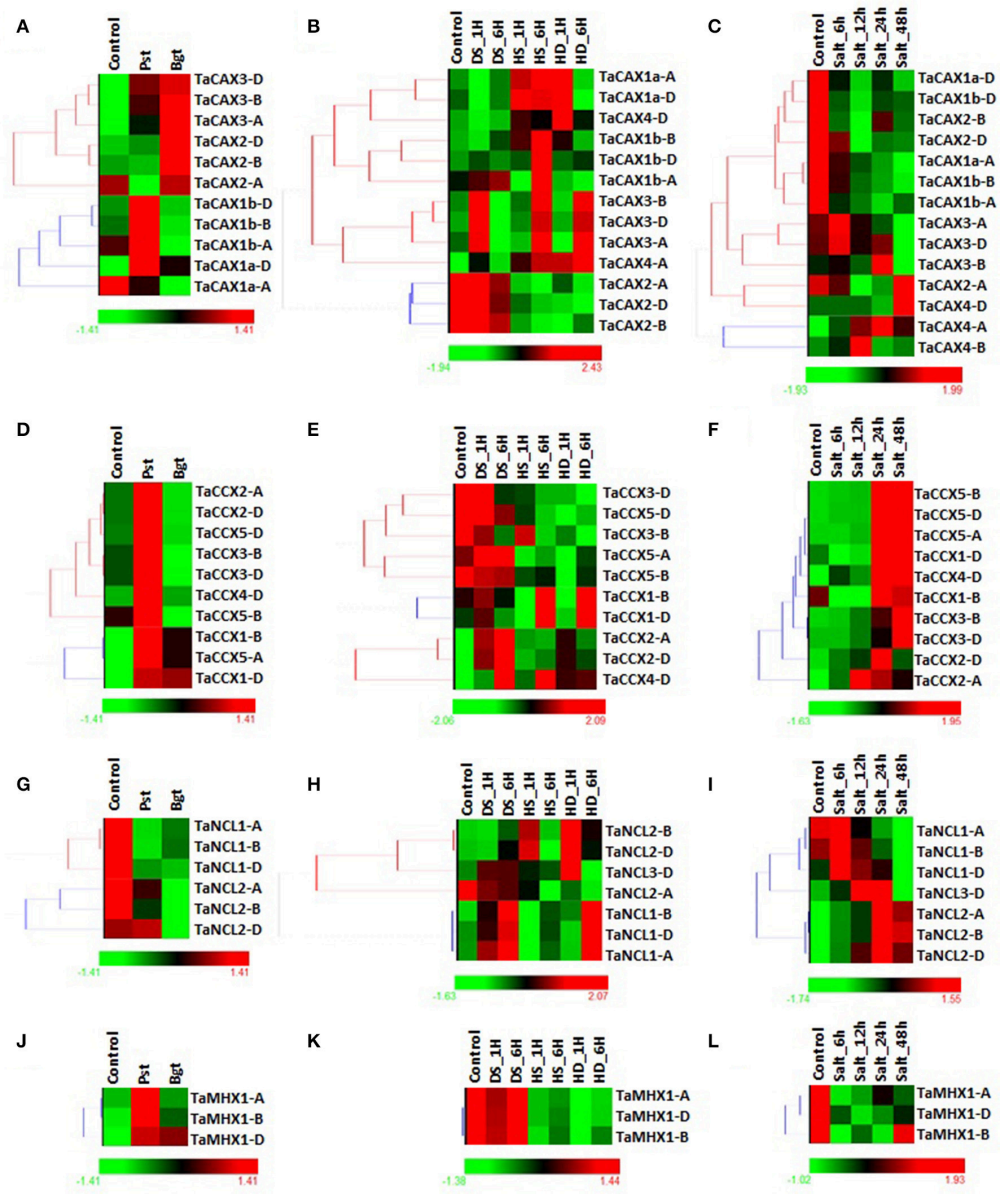
**Differential expression analysis of ***TaCaCA*** superfamily genes under biotic and abiotic stresses**. The heat map shows differential expression pattern of (1) *TaCAX* genes under **(A)** biotic, **(B)** heat/drought, and **(C)** salt stress, (2) *TaCCX* genes under **(D)** biotic, **(E)** heat/drought and **(F)** salt stress **(F)**, (3) *TaNCL* genes under **(G)** biotic, **(H)** heat/drought and **(I)** salt stress, and (4) *TaMHX* genes under **(J)** biotic, **(K)** heat/drought stress and **(L)** salt stress. The symbols shown in figure indicate-HS; heat stress, DS; drought stress, HD; combination of heat and drought stress, Bgt; after *Blumeria graminis* infection, Pst; after *Puccinia striiformis* inoculation.

Various abiotic and biotic stresses affect numerous biochemical and physiological pathways in plants and ultimately responsible for yield loss (Munns and Tester, 2008; Prasad et al., 2011; Pradhan et al., 2012; Izadi et al., 2014). The calcium signaling has been reported as an important mechanism for these kinds of stress sensing in plants (Tuteja and Sopory, 2008; Kader and Lindberg, 2010; Zhang et al., 2014a). Modulation in expression of *CaCA* superfamily genes such as *CAXs* and *NCL* has been earlier reported during abiotic stresses in various plant species (Wang et al., 2012; Li et al., 2016; Pittman and Hirschi, 2016b). Therefore, it becomes necessary to study the impact of these stresses on expression pattern of *TaCaCA* genes. In the present study, we analyzed the effect of fungal pathogens (*Blumeria graminis;* Bgt, and *Puccinia striiformis*; Pst) under biotic stress, and heat, drought and salt under abiotic stress (Figure 7; Files S10–S12).

The expression of *TaCAX* genes were not much affected after fungal infestation. Only *TaCAX2* group genes were slightly (~2–3-folds) up-regulated (Figure 7A; File S10). The results indicated that the *CAX* genes are probably not much involved in fungal stress response. The *TaCAX4* group genes were found highlyS up-regulated during heat, drought and combination of these stresses, while *TaCAX1b-B* and *TaCAX1b-D* were up-regulated solely during heat stress (Figure 7B; File S11). Comparable expression of orthologous *OsCAX4* (LOC_Os02g04630) and *OsCAX1b* (LOC_Os05g51610) genes of rice, and *AtCAX5* (AT1G55730) and *AtCAX2* (AT3G13320) genes of Arabidopsis has been observed during similar stresses (Singh et al., 2015). During salt stress, *TaCAX1a-A, TaCAX1a-D, TaCAX1b-A*, and *TaCAX1b-D* were highly down-regulated, while *TaCAX4* group genes were up-regulated (Figure 7C; File S12). Modulated expression of numerous *CAX* genes has been reported in various plants under salt stress (Shigaki and Hirschi, 2006; Mei et al., 2007; Manohar et al., 2011; Han et al., 2012; Yamada et al., 2014). The expression pattern and role of *CAX* genes during different stresses has been recently listed from several plant species (Bickerton and Pittman, 2015), however the specific function of individual *TaCAX* genes is required to be established.

A total of six *TaCCX* genes showed differential expression during biotic stress. Majority of them were 2–3-folds up-regulated after Pst infestation, while two (*TaCCX2-A* and *TaCCX3-D*) were down regulated after Bgt infestation (Figure 7D; File S10). Likewise, the expression of orthologous *CCX* genes of Arabidopsis (AtCCX1; AT5G17860, AtCCX2; AT5G17850) are found up-regulated after wounding (Singh et al., 2015), which is usually considered as similar to pathogen infection. Heat, drought and their combination stresses modulated the expression of each *TaCCX* gene (Figure 7E; File S11), which indicated their role in these conditions. The *TaCCX2* and *TaCCX4* group genes were 2–18-folds up-regulated in different conditions. The *TaCCX4-D* was highly up-regulated in each stress. The *TaCCX1, TaCCX3* and *TaCCX5* group genes were 2–17-folds down-regulated in one or more stress condition. The *TaCCX3-D* was extremely down-regulated gene during all the stresses. Modulation of *CCX* genes expression during heat and drought stress is also reported in other plant species. For instance, AtCCX1 (AT5G17860) and AtCCX2 (AT5G17850) of Arabidopsis are up-regulated in similar stresses, while OsCCX4 (LOC_Os12g42910) is up-regulated in dehydration and down-regulated in heat stress (Singh et al., 2015). During exposure to the high salt conditions, majority of the *TaCCX* genes were 3–110-folds up-regulated. The *TaCCX2-D* (9–58-folds) and *TaCCX3-D* (11–110-folds) were highly affected genes at various stages of treatment (Figure 7F; File S12). The results indicated that the *TaCCX* genes were more affected during various abiotic stresses as compared to the other *TaCaCA* superfamily genes. The *CCX* genes are also found up-regulated during salt stress in rice and Arabidopsis (Morris et al., 2008; Singh et al., 2015).

Expression of *TaNCL* genes were also affected during various biotic and abiotic stresses as reported in other plant species (Wang et al., 2012; Singh et al., 2015). The *TaNCL2* group genes were found 2–8-folds down-regulated during both Pst and Bgt infestation, while *TaNCL1* and *TaNCL3* were not much affected (Figure 7G; File S10). *TaNCL2-A* and *TaNCL2-B* were highly (~8-folds) down-regulated during Pst and Bgt invasion, respectively. Variation in expression of *AtNCL* (AT1G53210) is also observed during wounding (Singh et al., 2015), which indicated role of *NCL* genes in pathogen response. Both *TaNCL1* and *TaNCL2* group genes showed assorted expression during heat, drought and their combination stresses (Figure 7H; File S11). Such as *TaNCL1* group genes were up-regulated during drought, while down-regulated during heat stress. The *TaNCL2-A* was down-regulated in most of the treatment, while *TaNCL2-B* and *TaNCL2-D* were found up-regulated. These results indicated functional divergence in *TaNCL* genes, despite of their similar structural organization. Moreover, the *AtNCL* (AT1G53210) and *OsNCL1* (LOC_Os01g11414) genes were up-regulated during heat stress in Arabidopsis and rice, respectively (Singh et al., 2015). During salt stress, *TaNCL2* group genes were (2–17-folds) up-regulated during all the treatments, in which *TaNCL2-B* was highly up-regulated in most of the treatments (Figure 7I; File S12). *TaNCL3-D* showed slight up-regulation till 24 h of treatment, while down-regulated at 48 h. Both *TaNCL1-A* and *TaNCL1-B* were ~3-folds down-regulated at 48 h of treatment. Increased expression of *AtNCL* and *OsNCL1* genes has also been reported in earlier studies (Wang et al., 2012; Singh et al., 2015).

The expression of *TaMHX* genes was not significantly affected during biotic stress; but these were 2–4-folds down-regulated during heat, drought and salt stresses (Figure 7J–L; Files S10–S12). However, the up-regulation of orthologous *OsMHX* (LOC_Os11g43860) and *AtMHX* (AT2G47600) genes is reported during similar stress conditions (Singh et al., 2015). The results indicated that the similar genes from different plants might behave differently during comparable stress conditions.

## Conclusions

Transporter proteins are an integral part of an organism to exchange the various mineral or metal ions. The Ca^2+^ ions play vital role in numerous development and defense related mechanisms in various plant species. The CaCA superfamily proteins are a kind of transporters involved in exchange of various metal ions including Ca^2+^ ion homeostasis. In the present study, we identified 34 TaCaCA superfamily proteins in *T. aestivum*, which were further classified into TaCAX (14 genes), TaCCX (10 genes), TaNCL (7 genes), and TaMHX (3 genes) families on the basis of their structural organization and phylogenetic relation with known proteins from other plant species. Similar to the other gene families in *T. aestivum*, the *TaCaCA* genes were also derived from each A, B, and D sub-genome and chromosome. The predicted homeologous genes showed similar gene and protein structure organization in each TaCaCA family. The hydropathy analysis indicated the occurrence of 10, 11, 9, and 9 TMs in TaCAX, TaCCX, TaNCL, and TaMHX proteins, respectively. Most of the TaCaCA proteins consisted of two Na_Ca_ex domains except TaNCL2 and TaNCL3 group proteins, which had single Na_Ca_ex domain. The TaNCL proteins comprised an additional EF-hand domain in CL region with conserved calcium binding motifs. As reported in other organisms, two α-repeats with specifically conserved signature motifs were also detected in each family of TaCaCA superfamily except TaNCL, which contained single α-repeat. The TaCAXs and TaCCXs were further classified into two (Type 1A and Type 1B) and three (group 1, 2, and 3) groups on the basis of similarity with the earlier known proteins, respectively. The significant expression of each *TaCaCA* gene during various developmental stages suggested their role in growth and development. The *TaCAX1a* and *TaCAX1b* group genes were specifically highly expressed in grain, while *TaNCL1* in root developmental stages. Modulated expression of certain *TaCaCA* genes during various biotic and abiotic stresses revealed their role in stress response. The *TaCCX* and *TaNCL* family genes were found more responsive than *TaCAXs* and *TaMHXs* during various abiotic stresses. The present study enlightened various characteristic features of CaCA superfamily proteins in *T. aestivum*, however the function of individual gene needs to be investigated using functional genomics tools in future studies.

## Author contributions

SU conceived the idea and designed the experiments. MT, ST, and SS performed the experiments. MT and SU analyzed the data, and wrote the manuscript.

### Conflict of interest statement

The authors declare that the research was conducted in the absence of any commercial or financial relationships that could be construed as a potential conflict of interest.

## Supplementary material

Figure S1 **Phylogenetic clustering of putatively identified CaCA superfamily proteins**. The tree was constructed using full length sequences of putatively identified CaCA proteins from *T. aestivum, T. urartu*, and *Aegilops tauschii* with the reported sequences from rice and arabidopsis. The CAX, CCX, NCL, and MHX family proteins are shown with red, purple, green, and blue color lines and fonts, respectively. Tight phylogenetic clustering confirmed the identity of isolated CaCA proteins from these plants.

Figure S2 **Multiple sequence alignment of CAX proteins**. The amino acid sequences of CAX proteins from *T. aestivum, Arabidopsis thaliana*, and *Oryza sativa* are aligned. Type 1A and Type 1B group of CAX proteins are separated by green line. The α1 and α2-repeat regions are shown in black boxes. The reported signature motif (GNxxE) in α-repeat regions (Kamiya and Maeshima, 2004; Cai and Lytton, 2004a) is indicated by green shaded region. The predicted transmembrane (TM1 to TM11) spans are over-lined in blue. The other conserved residues reported in CaCA superfamily proteins (Cai and Lytton, 2004a) are indicated by asterisk. Hyphens indicate gaps introduced to maximize the alignment. The identified Na_Ca_ex domains are shown as red shaded regions in both N- and C-terminus half of CAX proteins.

Figure S3 **Multiple sequence alignment of CCX proteins**. The amino acid sequences of CCX proteins from *T. aestivum, A. thaliana*, and *O. sativa* are aligned. The group 1, 2, and 3 CCX sequences are separated by green line. Conserved α1 and α2-repeat regions are shown in black colored boxes. The signature motifs “GNG(A/S)PD” in α1-repeat and “G(N/D)SxGD” in α2-repeat motifs (Cai and Lytton, 2004a) are shown by green shaded region. The predicted transmembrane (TM) spans (TM1 to TM11) are over-lined in blue color. The identified Na_Ca_ex domains are shown as red shaded regions in both N- and C-terminus half of CCX proteins.

Figure S4 **Multiple sequence alignment of NCL proteins**. The amino acid sequences of NCL proteins from *T. aestivum, A. thaliana*, and *O. sativa* are aligned. The α-2 repeat region and EF-hand domain is shown in black and red boxes, respectively. Calcium binding sites within EF-hand domain are indicated by pink asterisks. The Na_Ca_ex domains are shaded red. The predicted transmembrane spans (TM1 to TM11) are over-lined in blue.

Figure S5 **Multiple sequence alignment of CAX and NCL proteins**. The amino acid sequences of CAX and NCL proteins from *T. aestivum, A. thaliana*, and *O. sativa* are aligned together. The α-1 and α-2 repeat regions of are boxed in black while the signature motif (GNxxE) in dark red. The EF-hand domain is shown green box. Figure shows that NCL proteins lack α-1 repeat region and signature motif of CAX proteins.

Figure S6 **Multiple sequence alignment of NCL and NCX proteins**. The amino acid sequences of *T. aestivum* NCL proteins and human NCX proteins are aligned. The calcium binding domain of HsNCX proteins is over-lined in yellow, EF-hand domain of TaNCLs is red under-lined. The calcium binding sites in EF-hand domain is shown by red asterisks. Figure shows large variation between long (~230 AAs) calcium binding domain of HsNCXs and short (~65 AAs) EF-hand domain of TaNCL proteins.

Figure S7 **Multiple sequence alignment of MHX and NCX proteins**. The amino acid sequences of MHX proteins from *T. aestivum, A. thaliana*, and *O. sativa* and NCX proteins from human are aligned. The α-1 and α-2 repeat regions are boxed in black. Te predicted transmembrane (TM1 to TM10) spans are over-lined in blue, while Calcium binding domain HsNCX is yellow over-lined. Residues involved in Na^+^/Ca^2+^ exchange in HsNCX1 are indicated by asterisks, while the red asterisks indicates the residues which are not conserved in the MHX proteins. The Na_Ca_ex domains are shown as red shaded regions in both N- and C-terminus half of proteins.

File S1 **List of protein sequences identified as putative CaCA proteins from various databases**. Green highlighted sequences showed comparable size and hydropathy to the reported sequences, therefore these were used during further characterization.

File S2 **Clustering of *TaCaCA* superfamily genes into homeologous groups**. The genes derived from homeologous chromosomes and showed ≥90% similarity are considered as homeologous genes.

File S3 **Similarity matrix between various families of *TaCaCA* superfamily genes obtained by bi-directional best blast hit approach**. The genes highlighted with same color are predicted as putatively duplicated genes in each gene family due to their localization on same subgenome and relatively higher similarity than other genes.

File S4 **Analysis of chromosomal localization, gene structure and various other characteristic features of *TaCaCA* superfamily genes and proteins**.

File S5 **Number of transmembrane domains predicted by various tools in TaCaCA superfamily proteins**.

File S6 **Cytoplasmic loop sizes of various TaCaCA superfamily proteins**.

File S7 **Prediction of sub-cellular localization of TaCaCA superfamily proteins using various tools**.

File S8 **Domain organization in TaCaCA superfamily proteins**. Each TaCaCA protein was individually search against Pfam, NCBI-conserved domain database, SMART, Prosite-Scan and InterProScan servers to analyse the domain architecture.

File S9 **Expression analysis of *TaCaCA* genes in three developmental stages of various tissues**. The top expressing genes from various families are highlighted in red boxes.

File S10 **Fold expression change in *TaCaCA* superfamily genes during biotic stress of *Blumeria graminis* (Bgt) and *Puccinia striiformis* (Pst)**. Two-folds or more up and down regulated genes are shown in yellow and red color cells, respectively.

File S11 **Fold expression change in *TaCaCA* superfamily genes during drought (DS), heat (HS) and their combination (HD) stress**. Two-folds or more up and down regulated genes are shown in yellow and red color cells, respectively.

File S12 **Fold expression change in *TaCaCA* superfamily genes during salt stress**. Two-folds or more up and down regulated genes are shown in yellow and red color cells, respectively.
