# Supplementary figures and images for: Ca2+/Cation Antiporters (CaCA): Identification, Characterization and Expression Profiling in Bread Wheat (Triticum aestivum L.)
Source: Front Plant Sci. 2016 Nov 28;7:1775. doi: 10.3389/fpls.2016.01775 (PMC5124604; doi:10.3389/fpls.2016.01775)

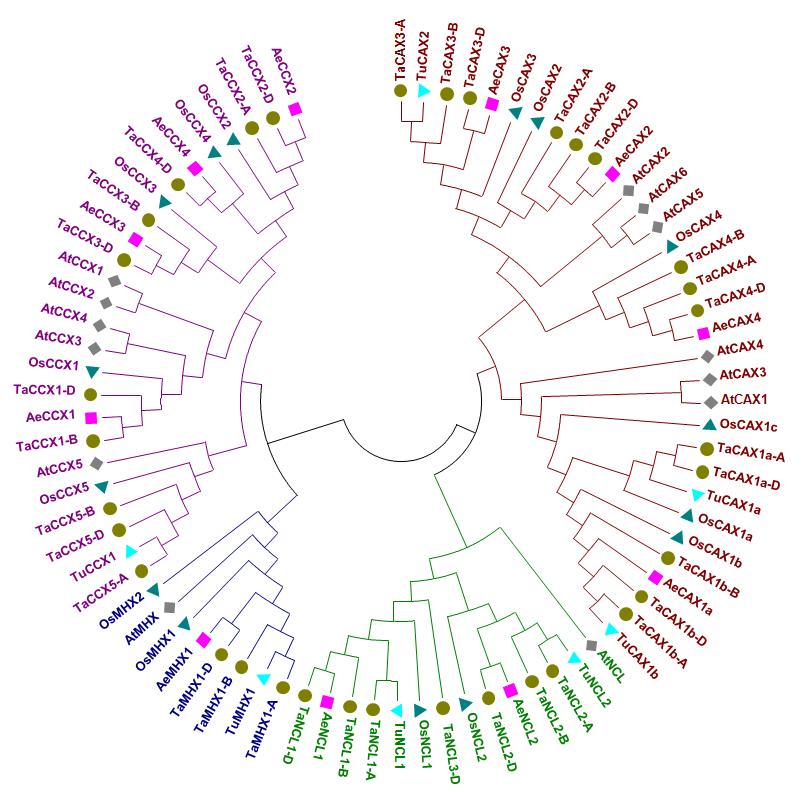

Supplement: Figure S1 — Phylogenetic clustering of putatively identified CaCA superfamily proteins. The tree was constructed using full length sequences of putatively identified CaCA proteins from T. aestivum, T. urartu, and Aegilops tauschii with the reported sequences from rice and arabidopsis. The CAX, CCX, NCL, and MHX family proteins are shown with red, purple, green, and blue color lines and fonts, respectively. Tight phylogenetic clustering confirmed the identity of isolated CaCA proteins from these plants. [file Image1.JPEG]

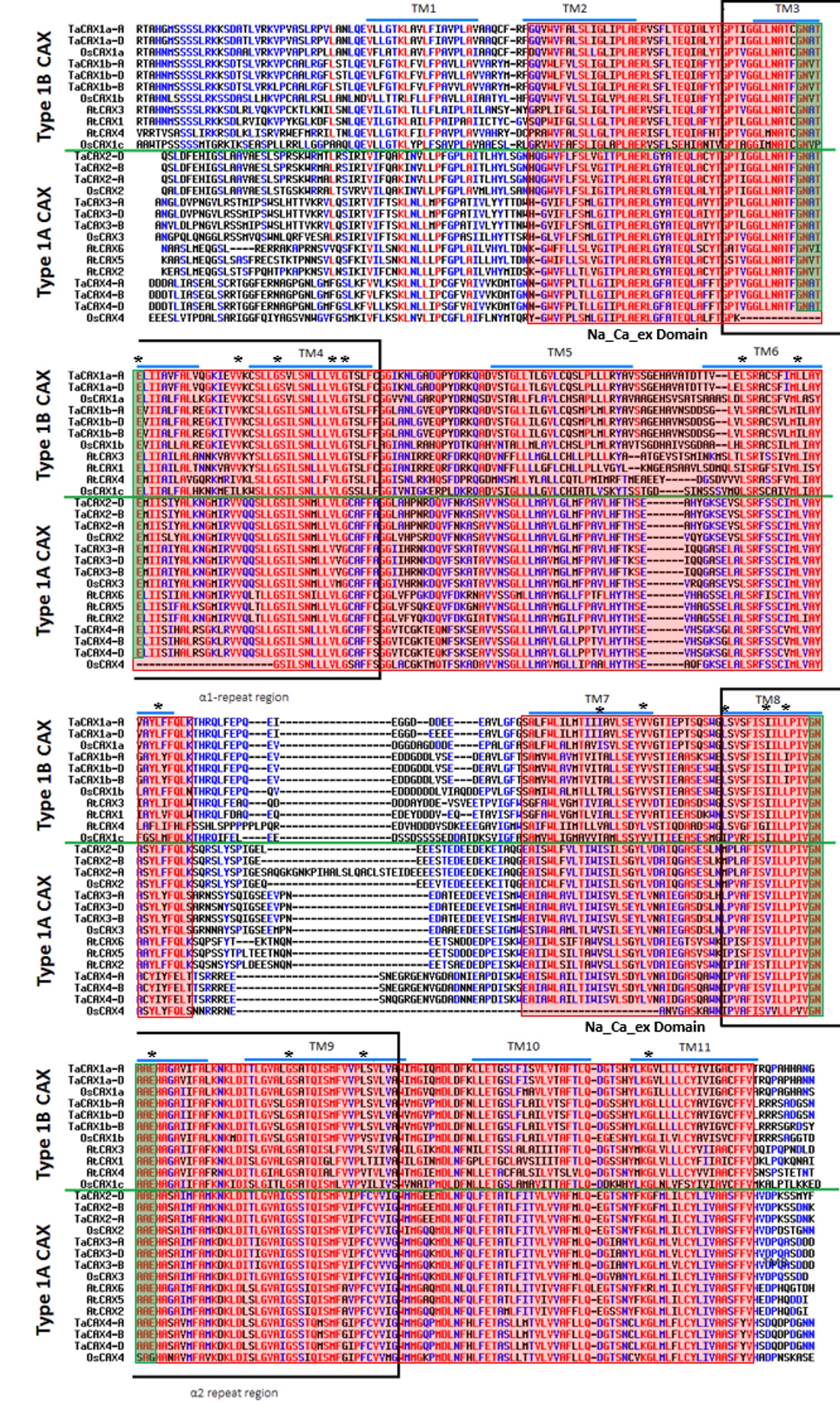

Supplement: Figure S2 — Multiple sequence alignment of CAX proteins. The amino acid sequences of CAX proteins from T. aestivum, Arabidopsis thaliana, and Oryza sativa are aligned. Type 1A and Type 1B group of CAX proteins are separated by green line. The α1 and α2-repeat regions are shown in black boxes. The reported signature motif (GNxxE) in α-repeat regions (Kamiya and Maeshima, 2004; Cai and Lytton, 2004a) is indicated by green shaded region. The predicted transmembrane (TM1 to TM11) spans are over-lined in blue. The other conserved residues reported in CaCA superfamily proteins (Cai and Lytton, 2004a) are indicated by asterisk. Hyphens indicate gaps introduced to maximize the alignment. The identified Na_Ca_ex domains are shown as red shaded regions in both N- and C-terminus half of CAX proteins. [file Image2.JPEG]

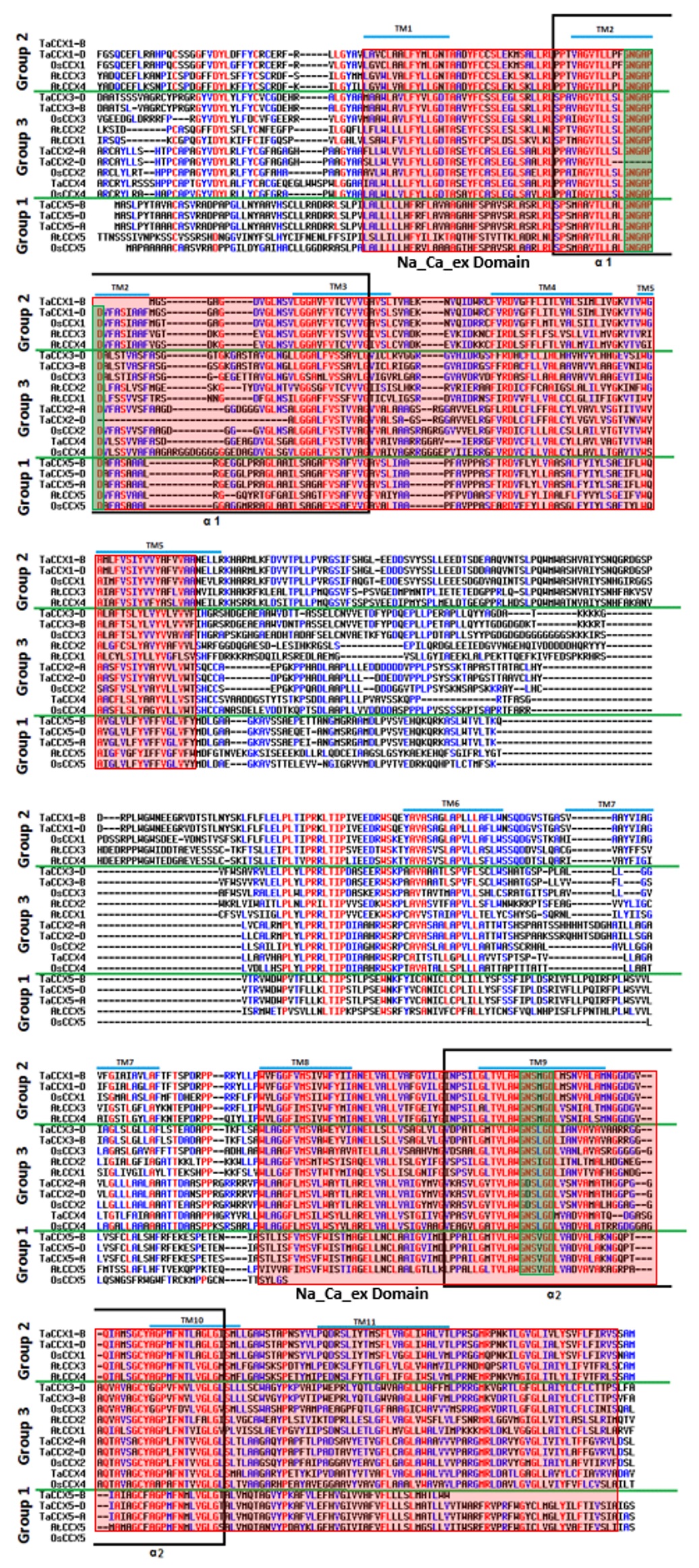

Supplement: Figure S3 — Multiple sequence alignment of CCX proteins. The amino acid sequences of CCX proteins from T. aestivum, A. thaliana, and O. sativa are aligned. The group 1, 2, and 3 CCX sequences are separated by green line. Conserved α1 and α2-repeat regions are shown in black colored boxes. The signature motifs “GNG(A/S)PD” in α1-repeat and “G(N/D)SxGD” in α2-repeat motifs (Cai and Lytton, 2004a) are shown by green shaded region. The predicted transmembrane (TM) spans (TM1 to TM11) are over-lined in blue color. The identified Na_Ca_ex domains are shown as red shaded regions in both N- and C-terminus half of CCX proteins. [file Image3.JPEG]

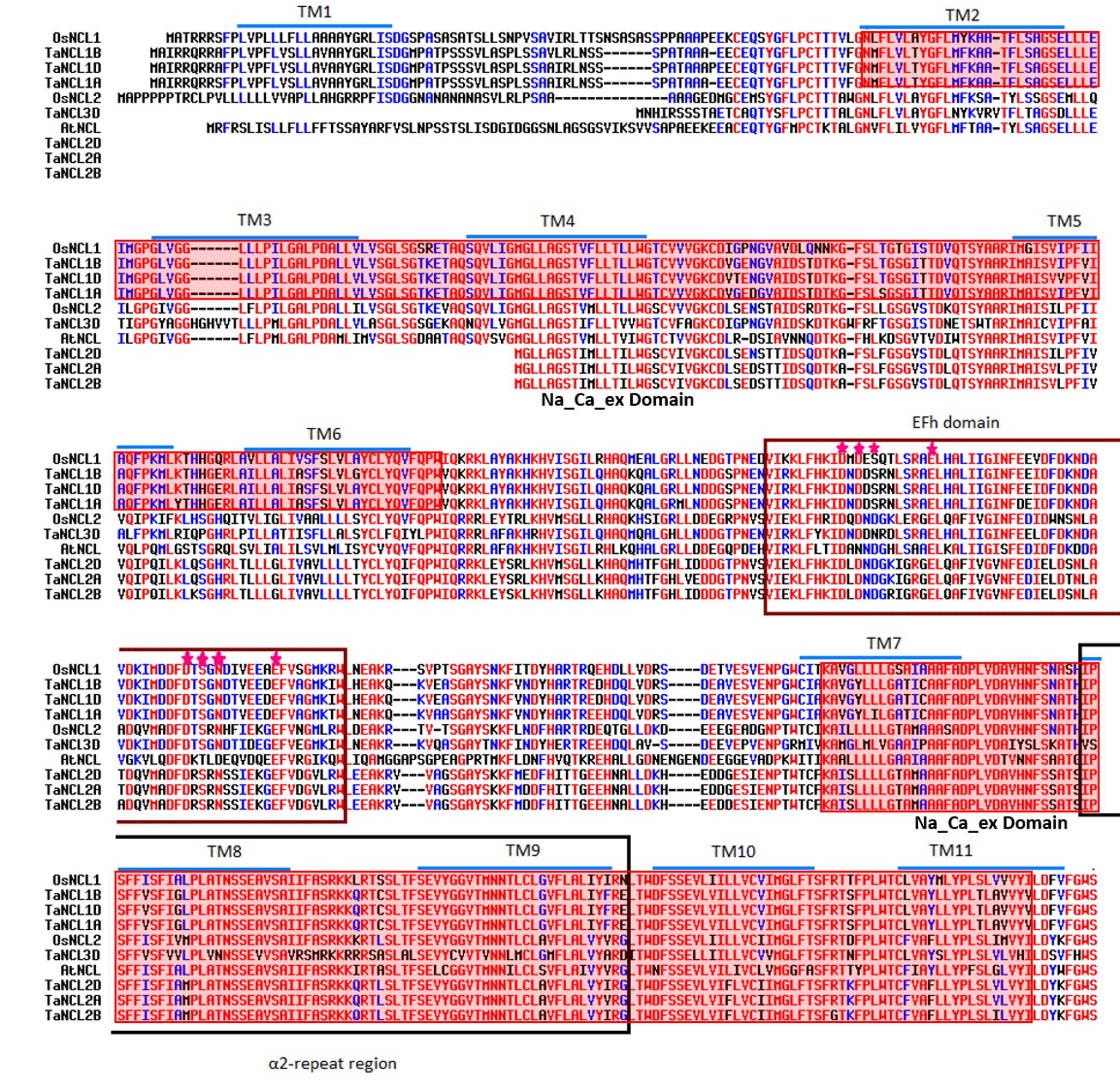

Supplement: Figure S4 — Multiple sequence alignment of NCL proteins. The amino acid sequences of NCL proteins from T. aestivum, A. thaliana, and O. sativa are aligned. The α-2 repeat region and EF-hand domain is shown in black and red boxes, respectively. Calcium binding sites within EF-hand domain are indicated by pink asterisks. The Na_Ca_ex domains are shaded red. The predicted transmembrane spans (TM1 to TM11) are over-lined in blue. [file Image4.JPEG]

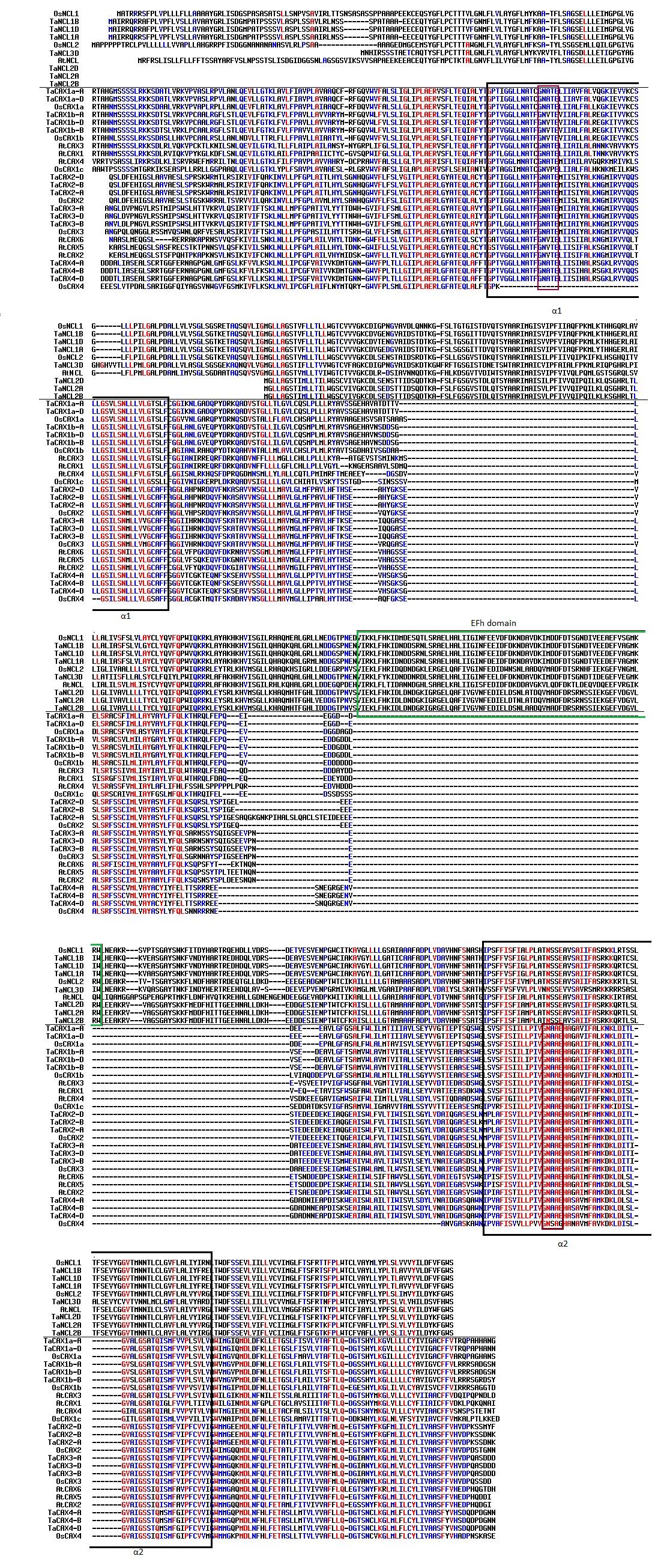

Supplement: Figure S5 — Multiple sequence alignment of CAX and NCL proteins. The amino acid sequences of CAX and NCL proteins from T. aestivum, A. thaliana, and O. sativa are aligned together. The α-1 and α-2 repeat regions of are boxed in black while the signature motif (GNxxE) in dark red. The EF-hand domain is shown green box. Figure shows that NCL proteins lack α-1 repeat region and signature motif of CAX proteins. [file Image5.JPEG]

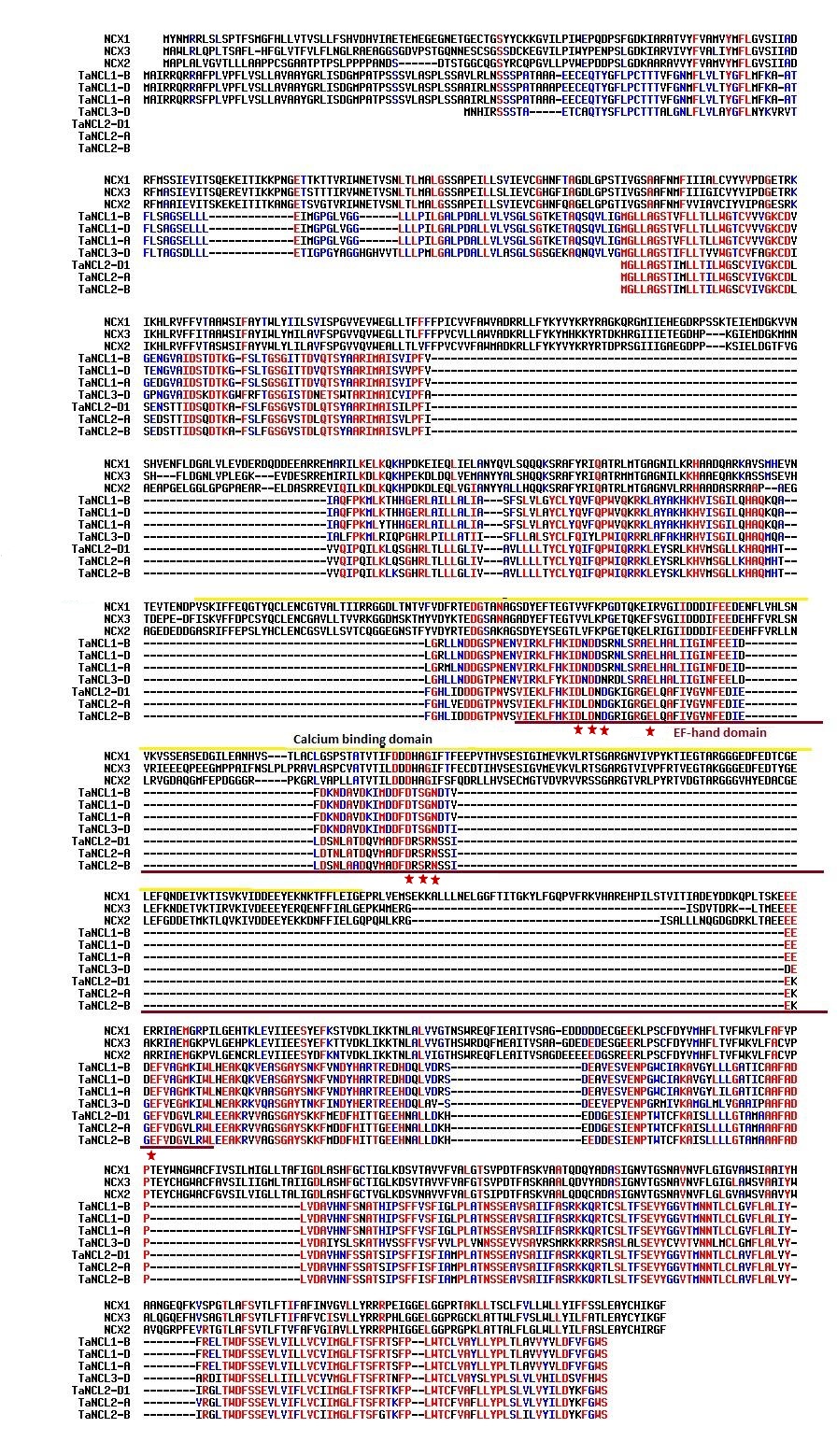

Supplement: Figure S6 — Multiple sequence alignment of NCL and NCX proteins. The amino acid sequences of T. aestivum NCL proteins and human NCX proteins are aligned. The calcium binding domain of HsNCX proteins is over-lined in yellow, EF-hand domain of TaNCLs is red under-lined. The calcium binding sites in EF-hand domain is shown by red asterisks. Figure shows large variation between long (~230 AAs) calcium binding domain of HsNCXs and short (~65 AAs) EF-hand domain of TaNCL proteins. [file Image6.JPEG]

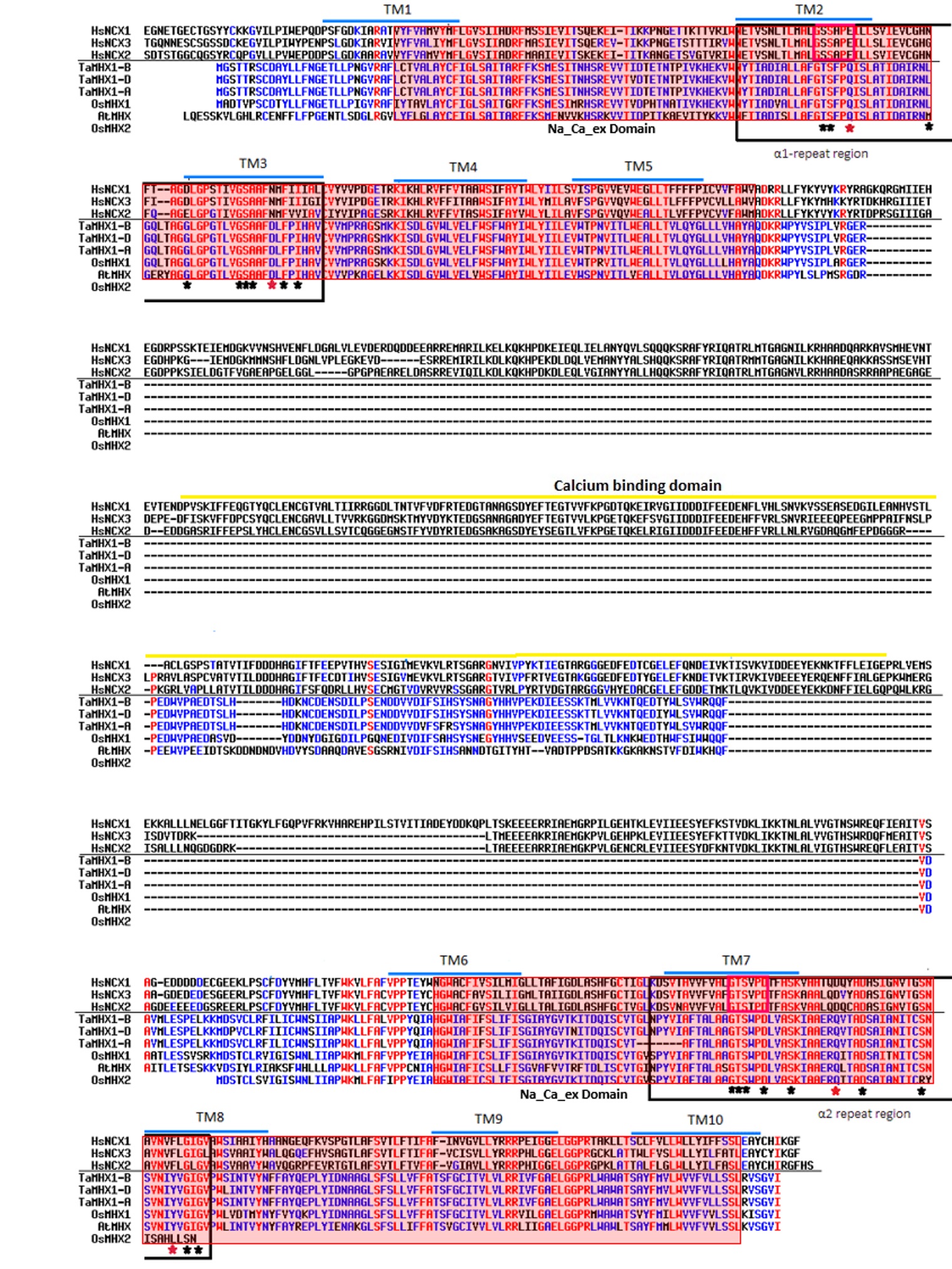

Supplement: Figure S7 — Multiple sequence alignment of MHX and NCX proteins. The amino acid sequences of MHX proteins from T. aestivum, A. thaliana, and O. sativa and NCX proteins from human are aligned. The α-1 and α-2 repeat regions are boxed in black. Te predicted transmembrane (TM1 to TM10) spans are over-lined in blue, while Calcium binding domain HsNCX is yellow over-lined. Residues involved in Na+/Ca2+ exchange in HsNCX1 are indicated by asterisks, while the red asterisks indicates the residues which are not conserved in the MHX proteins. The Na_Ca_ex domains are shown as red shaded regions in both N- and C-terminus half of proteins. [file Image7.JPEG]
